# Manipulating alternative end-joining alters carbon-ion beam-induced genome mutation profiles in *Arabidopsis thaliana*

**DOI:** 10.1093/dnares/dsaf014

**Published:** 2025-05-31

**Authors:** Jing Long, Jian Zhao, Jingmin Chen, Jianing Ding, Xiao Liu, Zhe Li, Po Bian, Ting Wang, Wenjie Jin, Xihong Lu, Yifan Zhang, Libin Zhou, Yan Du

**Affiliations:** Biophysics Group, Biomedical Center, Institute of Modern Physics, Chinese Academy of Sciences, Lanzhou 730000, China; University of Chinese Academy of Sciences, Beijing 100049, China; Biophysics Group, Biomedical Center, Institute of Modern Physics, Chinese Academy of Sciences, Lanzhou 730000, China; Biophysics Group, Biomedical Center, Institute of Modern Physics, Chinese Academy of Sciences, Lanzhou 730000, China; University of Chinese Academy of Sciences, Beijing 100049, China; Biophysics Group, Biomedical Center, Institute of Modern Physics, Chinese Academy of Sciences, Lanzhou 730000, China; University of Chinese Academy of Sciences, Beijing 100049, China; Biophysics Group, Biomedical Center, Institute of Modern Physics, Chinese Academy of Sciences, Lanzhou 730000, China; Biophysics Group, Biomedical Center, Institute of Modern Physics, Chinese Academy of Sciences, Lanzhou 730000, China; University of Chinese Academy of Sciences, Beijing 100049, China; Teaching and Research Section of Nuclear Medicine, School of Basic Medical Sciences, Anhui Medical University, Hefei 230032, China; Teaching and Research Section of Nuclear Medicine, School of Basic Medical Sciences, Anhui Medical University, Hefei 230032, China; Biophysics Group, Biomedical Center, Institute of Modern Physics, Chinese Academy of Sciences, Lanzhou 730000, China; Biophysics Group, Biomedical Center, Institute of Modern Physics, Chinese Academy of Sciences, Lanzhou 730000, China; Biophysics Group, Biomedical Center, Institute of Modern Physics, Chinese Academy of Sciences, Lanzhou 730000, China; Biophysics Group, Biomedical Center, Institute of Modern Physics, Chinese Academy of Sciences, Lanzhou 730000, China; University of Chinese Academy of Sciences, Beijing 100049, China; Heavy Ion Science and Technology Key Laboratory, Institute of Modern Physics, Chinese Academy of Sciences, Lanzhou 730000, China; Biophysics Group, Biomedical Center, Institute of Modern Physics, Chinese Academy of Sciences, Lanzhou 730000, China; University of Chinese Academy of Sciences, Beijing 100049, China

**Keywords:** Alt-EJ, carbon-ion beam, genome mutation, DNA damage response, genome stability

## Abstract

DNA double-strand breaks (DSBs) repair via POLQ-mediated alternative end-joining (Alt-EJ) is error-prone and mutagenic. However, Alt-EJ is often inhibited by classical nonhomologous end-joining (C-NHEJ) or homologous recombination, the precise impact of Alt-EJ on plant genome instability remains unclear. Here, we employed carbon-ion beam (CIB) which induce complex DSBs to bias cellular repair strategies toward Alt-EJ; additionally, a specific genetic background of C-NHEJ deficiency (*lig4-4*) *Arabidopsis thaliana* line and the POLQ-deficient (*teb-3* and *teb-8*) were combined to further amplify the mutagenic effects of CIB mediated by Alt-EJ. The *lig4-4* exhibited higher sensitivity to CIB than POLQ-deficient lines. *teb-8* exhibited constitutive DNA damage response (DDR), whereas DDR in *lig4-4* was strictly induced by CIB. At genome scale, *lig4-4* showed substantial changes in the insertion and deletion (InDels) mutation profile, with a higher proportion and larger size of InDels as well as greater microhomology dependence than wild-type. In contrast, *teb-8* showed moderate changes, including increased single-base InDels and complex mutations, but lacking > 30 bp InDels. Loss-of-function in LIG4 and POLQ resulted in a higher proportion of high-impact genome mutations than wild-type even at lower doses. These findings offered essential insights for the development of a novel repair pathway-driven heavy-ion beam mutagenesis system.

## 1. Introduction

The maintenance of genome stability and integrity is crucial for all organisms, as it not only underpins normal growth and development but also ensures the accurate transmission of genetic information to the offspring. Cells are constantly subjected to DNA damage caused by endogenous factors (such as reactive oxygen species, transposon activity, and replication stress) and exogenous stimuli (such as environmental pollution, extreme climates, pathogen infections, ultraviolet radiation, and ionizing radiation from human activities). Among these, double-strand breaks (DSBs) are the most severe and major causes of genome instability. If not repaired promptly or correctly, DSBs can lead to gene mutations, chromosomal aberrations, cell dysfunction, cell death, and disease.^1^ Thus, genome instability can be a double-edged sword; it elicits a negative response to stress but accumulates genetic mutations, which are important sources of biodiversity, evolution, adaptation, and mutagenesis.

Homologous recombination (HR) and classical nonhomologous end-joining (C-NHEJ) are the two main pathways involved in DSBs repair.^2,3^ HR is a relatively precise and high-fidelity repair mechanism that relies on homologous DNA sequences as templates and primarily functions in the G2 and S phases of the cell cycle.^4^ C-NHEJ functions throughout the cell cycle and has a higher repair efficiency than HR, making it the primary DSBs repair pathway in somatic cells of higher eukaryotes.^5^ It relies on the Ku70/80 heterodimer to recognize and bind to DSBs sites, utilizing XRCC4 and LIG4 to form a complex that rapidly re-ligates broken ends.^6–9^ In contrast to HR, C-NHEJ does not require homologous templates at the broken ends, although microhomologous sequences are sporadically used to accelerate the ligation. C-NHEJ is often imprecise and induces small mutations such as 0–4 bp InDels in genome.^10,11^

In addition to C-NHEJ, higher eukaryotic cells possess a genetically distinct non-homologous repair pathway known as alternative end-joining (Alt-EJ). Unlike C-NHEJ, Alt-EJ does not rely on Ku70/80 and LIG4, instead, it utilizes DNA polymerase θ (POLQ) to complete the final DNA end-joining repair.^12^ As Alt-EJ involves the use of microhomology sequences (2–20 bp) at the repair junctions, it is often referred to as microhomology-mediated end joining (MMEJ) in many literatures.^13^ POLQ, a structurally and functionally complex enzyme, is composed of three distinct domains: an N-terminal helicase-like domain, a large central domain, and a C-terminal polymerase domain.^14^ In *A. thaliana*, the POLQ is known as *TEBICHI* (*TEB*). Loss-of-function mutants of TEB exhibit increased sensitivity to genetic toxic agents, such as mitomycin C and methyl methanesulfonate,^15^ as well as defects in cell cycle progression.^16^ Studies on *Physcomitrella patens* and *Chlamydomonas reinhardtii* have confirmed their unique roles in the repair of CRISPR/Cas-induced DSBs.^17,18^ Beyond its role in Alt-EJ, POLQ has been implicated in base excision repair, translesion synthesis (TLS), and T-DNA integration.^14,19,20^ Although POLQ has been identified as key factors in Alt-EJ, the molecular mechanisms underlying Alt-EJ are not yet fully understood compared to those of C-NHEJ.

DSBs are the core mechanism for expanding genetic diversity through gene editing and heavy-ion beam irradiation technologies.^21–24^ Therefore, it is feasible to manipulate DSBs repair pathways to regulate the mutation spectrum and gene editing results. Currently, this strategy has been validated in multiple studies about genome editing.^21,22^ Dual loss of human POLQ and LIG4 showed 100% gene-targeting efficiency by abolishing random integration.^23^ However, inhibiting the specific DSBs repair pathway would alter the result of gene editing which can produce unintended outcomes. For instance, suppressing C-NHEJ resulted in an obvious increase of large deletions moderated by microhomology and plasmid integrations, these unintended outcomes may compromise the integrity of the genome.^24^ For this reason, enhancing the efficiency of gene editing by inhibition of key factors of DSBs repair remains controversial. In contrast to the site-directed mutagenesis of CRISPR/Cas9, the heavy-ion beam induces complex clustered DSBs across the entire genome to achieve random mutagenesis. Thus, manipulating DSBs repair pathways to explore the mutagenic potential of heavy-ion beam does not confront the same issues encountered in gene editing; conversely, it may facilitate the creation of novel mutational traits. Elucidating how tomodulate the heavy-ion beam-induced mutation spectra through the manipulation of DSBs repair mechanism holds significant theoretical and practical value for mutagenesis breeding.

Alt-EJ is of higher mutagenicity than C-NHEJ, as it frequently induces chromosomal rearrangements, larger insertions, and deletions (InDels).^25,26^ Therefore, it can serve as a critical target for modulating the mutation spectrum of heavy-ion beam irradiation. However, in previous studies, Alt-EJ is generally considered to be a backup repair pathway as is often inhibited by HR and C-NHEJ.^12^ Unlike animal cancer cells, which frequently exhibit defects in classical repair pathways, and some prokaryotic organisms, which naturally lack the C-NHEJ pathway, plants generally maintain intact repair mechanisms. Consequently, the significance of Alt-EJ in maintaining plant genome stability has often been underestimated. In mammalian cells, the repair of complex DSBs induced by high linear energy transfer (LET) radiation is highly dependent on Alt-EJ. The resected complex DSBs or the absence of homologous sister chromatid can both make a more dependent on Alt-EJ for DSBs repair.^27^ Therefore, to clarify the role of the Alt-EJ repair pathway in plant genome stability, it is crucial to introduce complex DSBs that can break the preference for repair pathways. Carbon-ion beam (CIB), a type of high LET ionizing radiation, deposits large amounts of energy densely along the particle track, resulting in clustered DNA damages via direct energy deposition and indirect oxidative damage through water radiolysis.^28^ In addition, a specific genetic background, such as C-NHEJ deficiency, can amplify the mutagenic effects of CIB mediated by Alt-EJ. In this study, the T-DNA insertion *A. thaliana* lines *teb* (which encodes POLQ) and *lig4* were exposed to CIB irradiation, after which radiosensitivity and DDR characteristics in the irradiation generation, as well as the frequency and spectrum of genomic variation in their offspring were comprehensively investigated. We aimed to clarify the role of Alt-EJ in the repair of CIB-induced DSBs and provide significant insights for advancing the development of novel repair pathway-driven heavy-ion beam mutagenesis systems.

## 2. Materials and methods

### 2.1. Plant materials and growing conditions

The WT *A. thaliana* used in this study was Columbia-0 (Col-0). The *lig4-4* (At5g57160, SALK_044027), *teb-3* (At4g32700, SALK_001669), and *teb-8* (At4g32700, SALK_200962) T-DNA insertion lines were obtained from Arashare (https://www.arashare.cn/index). Plant genotypes were identified by polymerase chain reaction (PCR) using the universal primer LB (left-border) for T-DNA insertion and gene-specific primers (Table S1). The *A. thaliana* plants were cultured in a growth room maintained at 23 °C with a photoperiod of 16 h of light and 8 h of darkness.

### 2.2. Genotoxicity testing

#### 2.2.1. CIB irradiation treatment

WT, *lig4-4*, and *teb* seeds were sandwiched between two layers of Kapton membrane, then placed in petri dishes (Φ60 mm) and irradiated using CIB (^12^C^6+^) generated at the Heavy Ion Research Facility in Lanzhou (HIRFL), Institute of Modern Physics, Chinese Academy of Sciences. The initial energy of CIB was 80 MeV/u, with a nominal LET of 30 keV/μm and a dose rate of 60 Gy/min. The irradiation doses for WT *A. thaliana* were 0, 100, 200, 300, and 350 Gy, whereas those for *teb-3*, *teb-8*, and *lig4-4* mutants were 0, 40, 80, 100, 120, and 160 Gy. The irradiated seeds (M_1_) were sown in the soil, and after 24 days of growth, phenotypic observations and rosette leaf area measurements were conducted. The seeds of M_1_ plants were harvested from individuals and recorded as M_2_. For seedlings irradiation, seeds were sterilized with 2% Plant Preservative Mixture (Yeasen, China, Cat: 41009ES25), and stratified at 4 °C in the dark for 2 days. All seedlings were grown on vertical plates for seven days in a growth room and then were closely attached to the surface of new 1/2 MS medium for CIB radiation at a dosage of 25 Gy with a nominal LET of 30 keV/μm.

#### 2.2.2. Root growth test under hydroxyurea (HU), bleomycin (zeocin), and CIB treatments

Seeds were sterilized and stratified as described in 2.2.1. For chemical reagent-responsive root elongation assays, sterilized seeds were sown on solid 1/2 MS medium (containing 1.5% sucrose and 0.4% Phytagel) supplemented with 0.5, 0.75, 1, and 1.25 mM HU (Solarbio, China, Cat: H8420) and 10, 50, 100, and 200 nM zeocin (Maokangbio, China, Cat: MS0009), as well as the control (without HU and zeocin). Sterilized seeds derived from CIB irradiation were sown directly on 1/2 MS medium. All seedlings were grown on vertical plates for 8 d in a growth room. Root length was measured using the ImageJ software (version 1.54j). Three biological replicates were used for each treatment.

### 2.3. RNA extraction and sequencing

Samples of WT, *lig4-4*, and *teb-8* seedlings were collected at 6 h post-irradiation. Total RNA was extracted from seedlings using an RNA Plant Extraction Kit (Cat: DP432; Tiangen, China). RNA purity and concentration were determined using a TECAN Infinite 200 PRO microplate reader (Tecan Life Sciences, Switzerland). RNA integrity was assessed using an RNA Nano 6000 Assay Kit on a Bioanalyzer 2100 system (Agilent Technologies, CA, USA). cDNA library construction and sequencing were performed by Novogene Bioinformatics Technology Co., Ltd. (China). RNA-Seq was performed in at least three biological replicates for each treatment. The quality and detailed information on the RNA-seq libraries for *teb-8* and *lig4-4* are shown in Table S2.

The raw data in the FASTQ format were initially processed using in-house Perl scripts. Clean data were obtained by filtering reads containing adapters, poly N sequences, and low-quality reads from the raw data. Gene expression levels were quantified using fragments per kilobase of exon per million fragments mapped (FPKM) method. DESeq2 was used to identify differentially expressed genes (DEGs), with a |log_2_ (Fold Change)| ≥ 1 and a Bonferroni-corrected *P*-value (p. adjust) < 0.05 as the screening criteria for differential expression analysis. The R package clusterProfiler (v4.10.0) was used for Gene Ontology (GO) annotation, Kyoto Encyclopedia of Genes and Genomes (KEGG), and enrichment analysis of DEGs.^29^ The pheatmap package (v1.0.12) was used to generate heatmaps.

### 2.4. Quantitative reverse-transcriptase polymerase chain reaction (qRT-PCR)

To validate the transcriptome sequencing results, we selected nine DEGs related to DDR for qRT-PCR validation using the primers provided in Table S3. Primers were designed using Primer3Plus (https://www.primer3plus.com) and synthesized by Sangon Biotech (China). cDNA was synthesized using the First Strand cDNA Synthesis Kit (Cat: KR118; Tiangen, China), and qRT-PCR was performed using the SYBR Fluorescence Quantification Kit (Cat: FP205; Tiangen, China). The PCR cycle included denaturation at 95 °C for 3 min, followed by 40 cycles at 95 °C for 15 s and 58 °C for 30 s. The expression of ACTIN2 (At3g18780) was used as an internal control. Each sample was tested in triplicate to ensure reliability. The relative gene expression was calculated using the 2^−ΔΔCt^ method.

### 2.5. Whole-genome resequencing and mutation validation

More than 10 M_2_ plants of WT (200 Gy), *teb-8* (100 Gy), and *lig4-4* (100 Gy) from different M_1_ individuals, as well as several unirradiated plants, were randomly selected. Genomic DNA was extracted from rosette leaves using the hexadecyltrimethylammonium bromide method. The integrity of the DNA samples was assessed by 1% gel electrophoresis, and the OD260/OD280 ratio was measured using a TECAN Infinite 200 PRO microplate reader (Tecan Life Sciences, Switzerland). High-quality DNA samples were used for whole genome resequencing. The samples were sent to Novozymes Bioinformatics Co., Ltd. (China), where DNA libraries were constructed and sequenced using the Illumina NovaSeq 6000 platform, with an average sequencing depth above 40×. The clean reads were mapped to the *A. thaliana* reference genome (TAIR10), and single base substitution (SBSs) and small InDels were identified using SAMtools and VarScan2 (v.3.9), according to the bioinformatics analysis protocol established in our previous studies.^30,31^ The mapping quality and detailed information of whole-genome resequencing are shown in Table S4. In each genotype, the common variants shared by two or more sequenced individuals were removed when the allele frequency of reads supporting variations was ≥ 25% in the target sample or < 5 % in other samples. Randomly selected mutation sites were validated using Integrative Genomics Viewer and Sanger sequencing. Primers for validation were designed using Primer3Plus (https://www.primer3plus.com/), and all primer sequences are listed in Table S5. The same DNA samples used for whole-genome resequencing were used as PCR templates, and successfully amplified products were sent to Sangon Biotech (China) for Sanger sequencing. Positive mutations were annotated using SnpEff v.4.2.^32^ To perform microhomology analysis, we extracted the flanking sequences of InDels mutations using TBtools-II.^33^ The structural variants (SVs) calling was performed by sv-callers, a Snakemake-based workflow that unites Lumpy, Delly and Manta, etc.^34^

### 2.6. Statistical analysis

One-way analysis of variance (ANOVA) and Duncan’s multiple-range test were conducted by using SPSS 26 to assess statistical significance. Statistical significance was set at *P *< 0.05. Graphs were generated using GraphPad Prism 10, Origin 2022 and R4.4, the distribution of genome mutations was visualized using the Advance Circos package of TBtools.^35^

## 3. Results

### 3.1. Loss functions in TEB and LIG4 exhibited distinct sensitivities to various genotoxic stresses

**Fig. 1.**
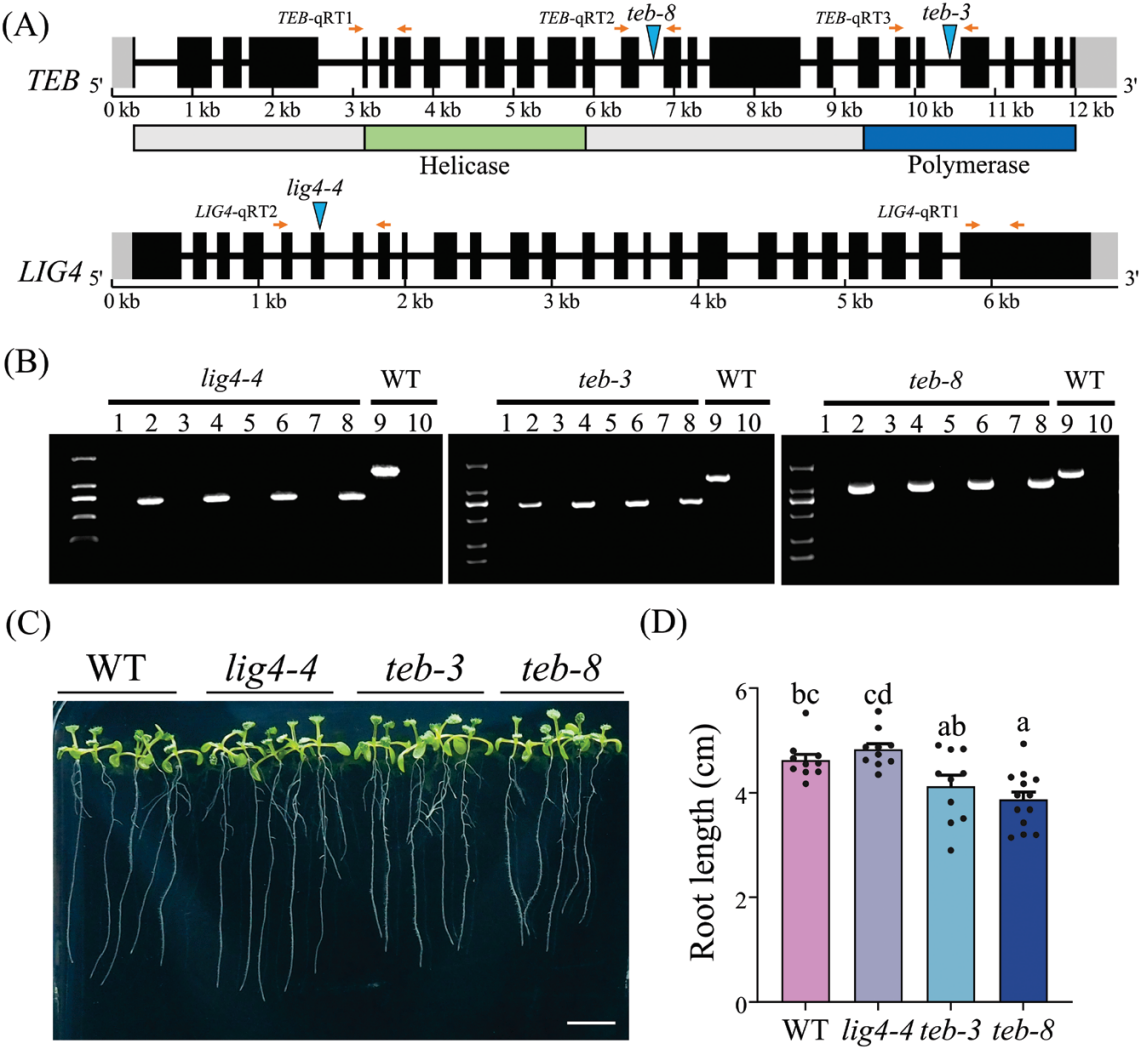
Validation and phenotypic analysis of *lig4* and *teb* mutants. (A) Structure of the *TEB* and *LIG4* genes and location of the T-DNA insertion in different alleles. Exons are indicated by black boxes and introns are indicated by black lines. Locations of the *teb-8*, *teb-3,* and *lig4-4* T-DNA insertion lines are marked. Five pairs of primers and their orientations are shown by arrows. Predicted TEB domains are shown under the gene structure. (B) Genotyping results for WT, *teb*, and *lig4* mutants. Homozygous T-DNA insertion mutants were identified using primers flanking the T-DNA insertion sites listed in Table S1. Odd-numbered lanes were identified using the corresponding LP and RP primers, while even-numbered lanes were identified using the LBb1.3 and corresponding RP primers. For each mutant line, four individual plants were randomly selected for verification, with lanes 1 and 2 representing the first plant, lanes 3 and 4 representing the second plant, and so on. (C) Representative images of WT and indicated mutant seedlings grown for 8 d. Bar = 1 cm. (D) Statistics of root length data. Different letters indicate significant differences among the different lines (means ± SEM, n > 10, *P* < 0.05, One-way ANOVA with Duncan’s method).

The *teb-3*, *teb-8*, and *lig4-4* mutant lines of *A. thaliana* (Fig. 1A) were used in this study. All three lines were confirmed to be homozygous using the three-primer method (Fig. 1B). In addition, the expression levels of target genes were investigated. Compared with the WT, the expression of *LIG4* was dramatically reduced in the *lig4-4* mutant, and no detectable transcripts were produced in the *teb-3* and *teb-8* mutants (Fig. S1A). Under normal growth conditions, no significant differences in root length were observed between *lig4-4* and WT plants, whereas both *teb-3* and *teb-8* plants exhibited significantly reduced root lengths (Fig. 1C–D).

**Fig. 2.**
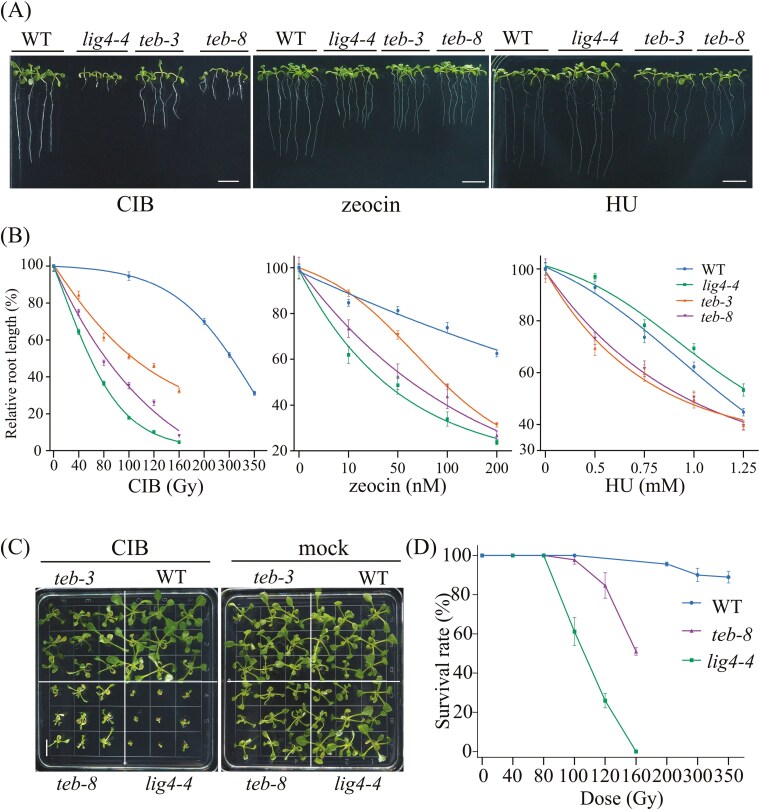
Sensitivity of WT, *lig4-4*, *teb-8*, and *teb-3* to multiple genotoxic stresses. (A) Representative images of roots from WT and mutant lines. CIB dose: 100 Gy, zeocin concentration: 100 nM, HU concentration: 1 mM. Seedlings were grown vertically on 1/2 MS medium for 8 days. Bar = 1 cm. (B) Quantitative analysis of relative root length in WT and mutant of seedlings after CIB exposure, zeocin, and hydroxyurea treatments (*n* > 30, means ± SEM), log(agonist) vs. response -- Variable slope (four parameters). (C) Phenotypes of WT and mutants with and without CIB irradiation at a dose of 100 Gy, cultured on 1/2 MS medium for two weeks. Bar = 1cm. (D) Survival rate of WT, *teb-8*, and *lig4-4* mutants after two weeks of culture in 1/2 MS medium (*n* > 35, means ± SEM).

We used CIB (primarily inducing clustered DNA damage), zeocin (primarily inducing DNA strand breaks), and HU (causing replication stress) to assess the root growth sensitivity of the three mutant lines and WT in response to different types of damage (Fig. 2A). Compared to the WT, all mutants exhibited hypersensitivity to CIB irradiation and zeocin, with a sensitivity ranking of *lig4-4* > *teb-8* > *teb-3* > WT (Fig. 2B). Only *teb-8* and *teb-3* showed hypersensitivity to HU, whereas *lig4-4* root growth showed lower sensitivity than the WT (Fig. 2A–B). These findings suggest that *teb-3 and teb-8* are hypersensitive to both DNA strand breaks damage and replication stress, whereas *lig4-4* exhibits specific hypersensitivity to DSBs.

Because the radiosensitivity of *teb-8* was higher than *teb-3*, only *teb-8* was used in further study. We further quantified the radiosensitivity of *lig4-4* and *teb-8* using multiple metrics, including survival rate, phenotypic observation, and leaf size measurements (Fig. 2C–D and Fig. S1B–D). The 50% lethal dose for *lig4-4* was approximately 100 Gy, whereas for *teb-8*, it was 160 Gy. Even at 350 Gy, the WT exhibited a relatively low lethality rate of 11.1% (Fig. 2D). Upon exposure to 100 Gy CIB radiation, *lig4-4* exhibited severe defects, including a dramatic reduction in root length, smaller plants, and more severe leaf deformation (Fig. 2A and C). These findings suggest that *lig4-4* is more sensitive to CIB than *teb-8*.

### 3.2. In seedling development phase, *teb-8* exhibited a constitutive DDR, whereas the DDR in *lig4-4* was strictly dependent on the induction of CIB irradiation

**Fig. 3.**
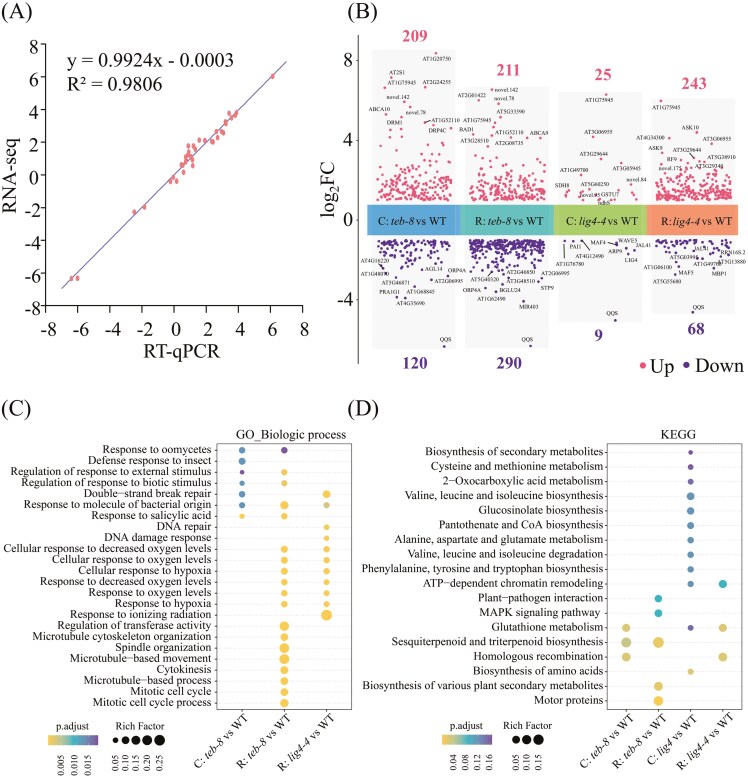
Overview of transcriptome responses to CIB irradiation in *teb-8* and *lig4-4*. (A) Correlation scatter plot of RT-qPCR experimental log_2_ (Fold Change) values and RNA-seq log_2_ (Fold Change) values for 9 DEGs. ‘C’ denotes the control group (without CIB treatment), ‘R’ signifies the irradiation treatment group. (B) Number of up- and down-regulated DEGs in different compare groups. (C) and (D) GO (biological processes only) and KEGG enrichment of DEGs in different compare groups. The size and color of the circles indicate the rich factor and significance value (p. adjust), respectively.

**Fig. 4.**
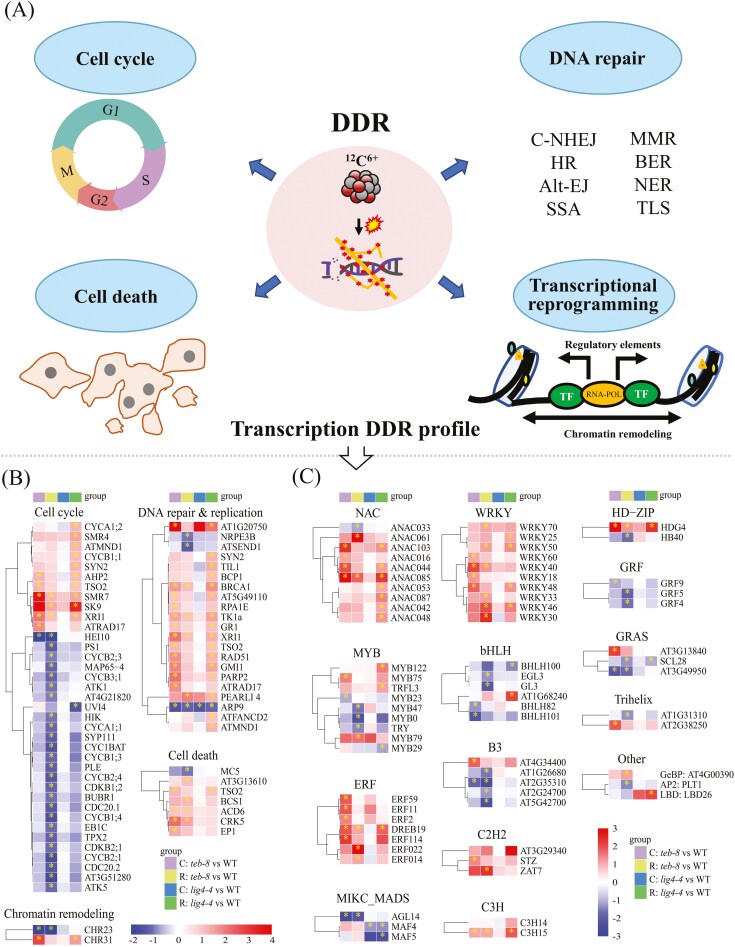
Transcription-level changes of DDR profile. (A) Schematic diagram of processes involved in DDR. (B) and (C) Heatmap of 61 DDR-related genes and 68 TFs among the 902 DEGs. The heatmap was created using log_2_ (Fold Change).

To analyze the molecular responses of *teb-8* and *lig4-4* to complex DNA damage, 8-day-old seedlings were exposed to CIB, followed by comparative transcriptomic analysis. A series of comparison groups were established, labeled as ‘C: *teb-8* vs WT’, ‘R: *teb-8* vs WT’, ‘C: *lig4-4* vs WT’, ‘R: *lig4-4* vs WT’, ‘WT: R vs C’, ‘*teb-8*: R vs C’, ‘*lig4-4*: R vs C’. Here, ‘C’ denotes the control group, whereas ‘R’ represents the irradiation treatment group. A strong correlation between the results of RT-qPCR and RNA-seq (*R*^2^ = 0.98) based on several DEGs indicated the reliability of our RNA-seq data (Fig. 3A). A total of 329 DEGs (209 and 120 with upregulated and downregulated expressions, respectively) were identified in ‘C: *teb-8* vs WT’, whereas only 34 DEGs (25 and 9 with upregulated and downregulated expressions, respectively) were found in ‘C: *lig4-4* vs WT’ (Fig. 3B). In the CIB irradiation treatment group, 501 (211 and 290 with upregulated and downregulated expressions, respectively) and 311 (243 and 68 with upregulated and downregulated expressions, respectively) DEGs were identified in ‘R: *teb-8* vs WT’ and ‘R: *lig4-4* vs WT’, respectively (Fig. 3B). Combined with subsequent GO and KEGG enrichment analysis, we noticed that for *teb-8*, even under the control group, the DSBs repair pathway was significantly enriched in ‘C: *teb-8* vs WT’, whereas for *lig4-4*, it was enriched only after CIB irradiation. These results confirmed that *teb-8* exhibited a constitutive DDR, whereas the DDR in *lig4-4* was strictly dependent on the induction of CIB irradiation (Fig.4B and Table S6). Furthermore, we also conducted a comparative analysis of DEGs between the ‘C: *teb-8* vs WT’ and ‘R: *teb-8* vs WT’. A total of 114 DEGs were shared between the two groups, while 215 and 387 DEGs were uniquely identified in the C: *teb-8* vs WT and R: *teb-8* vs WT, respectively. GO functional annotation of these three DEG categories revealed enrichment in DNA damage response (DDR)-related pathways across all groups (Fig. S2).

### 3.3. Loss of function in LIG4 and TEB altered the seedling DDR transcriptional response to CIB irradiation

Here, we primarily focused on DDR-related processes, such as the cell cycle, DNA replication, damage repair, cell death, and core regulatory elements (TFs) involved in transcriptional reprogramming (Fig. 4A). GO and KEGG annotations were used to sort the DEGs associated with DDR-related processes in the ‘C: *teb-8* vs WT’, ‘R: *teb-8* vs WT’, ‘C: *lig4-4* vs WT’ and ‘R: *lig4-4* vs WT’ comparison groups. The expression patterns of these genes were plotted on a heatmap using the R package ‘pheatmap’ with log_2_FC values (Fig. 4B). A total of 61 genes associated with cell cycle (37), cell death (7), and DNA replication and repair (21) were identified, some of which function in multiple biological processes (Fig. 4B). When focused on the DEGs in the radiated groups, we found that for cell cycle–related DEGs, the expressions of 10 of the 11 DEGs in ‘R: *lig4-4* vs WT’ were significantly upregulated, whereas those of 25 of the 28 DEGs in *teb-8* showed significant downregulation. More DNA repair–related DEGs genes were detected in ‘R: *lig4-4* vs WT’ (17) than that in ‘R: *teb-8* vs WT’ (7). Gene set enrichment analysis was also conducted to analyze the regulation of specific pathways using all detected genes (the thresholds of FPKM and log_2_ (Fold Change) were not considered) in the irradiated groups (Fig. S3). In both ‘R: *teb-8* vs WT’ and ‘R: *lig4-4* vs WT’, cell death, stress response–related, and other repair pathways exhibited an upregulation trend, whereas the motor protein–related genes showed a downregulation trend. In addition, the shared DEGs among ‘WT: R vs C’, ‘*teb-8*: R vs C’ and ‘*lig4-4*: R vs C’, the response patterns of genes related to the cell cycle, cell death and DNA repair showed a predominant pattern of significant upregulation, with only two genes (AT2G44580 and ATN/AT3G05330) exhibiting downregulation (Fig. S4).

Chromatin remodeling often facilitates DNA damage repair and is closely related to the regulation of gene expression.^36^ Here, we identified two DEGs involved in this process: CHR23 and CHR31. CHR23 expression was downregulated in both ‘C: *teb-8* vs WT’ and ‘R: *teb-8* vs WT’, and CHR31 expression was upregulated in both ‘C: *teb-8* vs WT’ and ‘R: *lig4-4* vs WT’. Transcription factors are central regulators of transcriptional reprogramming and are indispensable for various cellular processes, including modulation of gene expression, signal transduction, and responses to environmental stress. We searched for TFs in all 902 DEGs using the *A. thaliana* transcription factor database (PlantTFDB v5.0). A total of 68 TFs belonging to the transcription factor families WRKY (10), MYB (9), NAC (10), ERF (6), and bHLH (6) were identified (Fig. 4C). Among them, the expressions of TFs WRKY, NAC, ERF, and C2H2 were predominantly upregulated. The GO enrichment analysis of 68 TFs revealed associations with responses to various plant hormones (Fig. S5A).

### 3.4 Loss of function in LIG4 and TEB caused pronounced differences in genomic mutation accumulation in their offspring

DNA damage types and cellular repair strategies determine genomic variation profiles. To investigate the effects of CIB and different DNA repair states on the genomic mutation spectrum of the progeny, CIB-treated seeds (M_1_) were sown and M_2_ seeds were harvested from individual M_1_ plants. Seeds of the non-irradiated group were sown and harvested in the same manner as those of the CIB-treated group to synchronize genetic generations. Subsequently, the genome mutation frequency and spectrum were characterized by whole-genome resequencing of the M_2_ lines *teb-8*-100 Gy (11 lines), *lig4-4*-100 Gy (14 lines), and WT-200 Gy (10 lines) derived from different M_1_ individual plants. Additionally, control lines of WT (four lines), *teb-8* (five lines), and *lig4-4* (seven lines) that were not treated with CIB were included in the analysis. Randomly selected mutation sites were validated by Sanger sequencing (Fig. S5B). Based on our previous descriptions and definitions,^30,37^ the detected mutations were categorized into seven categories, including SBSs, single-base insertions (+ 1), single-base deletions (− 1), deletions in size above 2 bp (Dels ≥ 2 bp), insertions in size above 2 bp (Ins ≥ 2 bp), and complex mutations (two or more mutations occurring within 10 base pairs), and SVs.

#### 3.4.1 Differential distribution and frequency of SBSs and small InDels

**Fig. 5.**
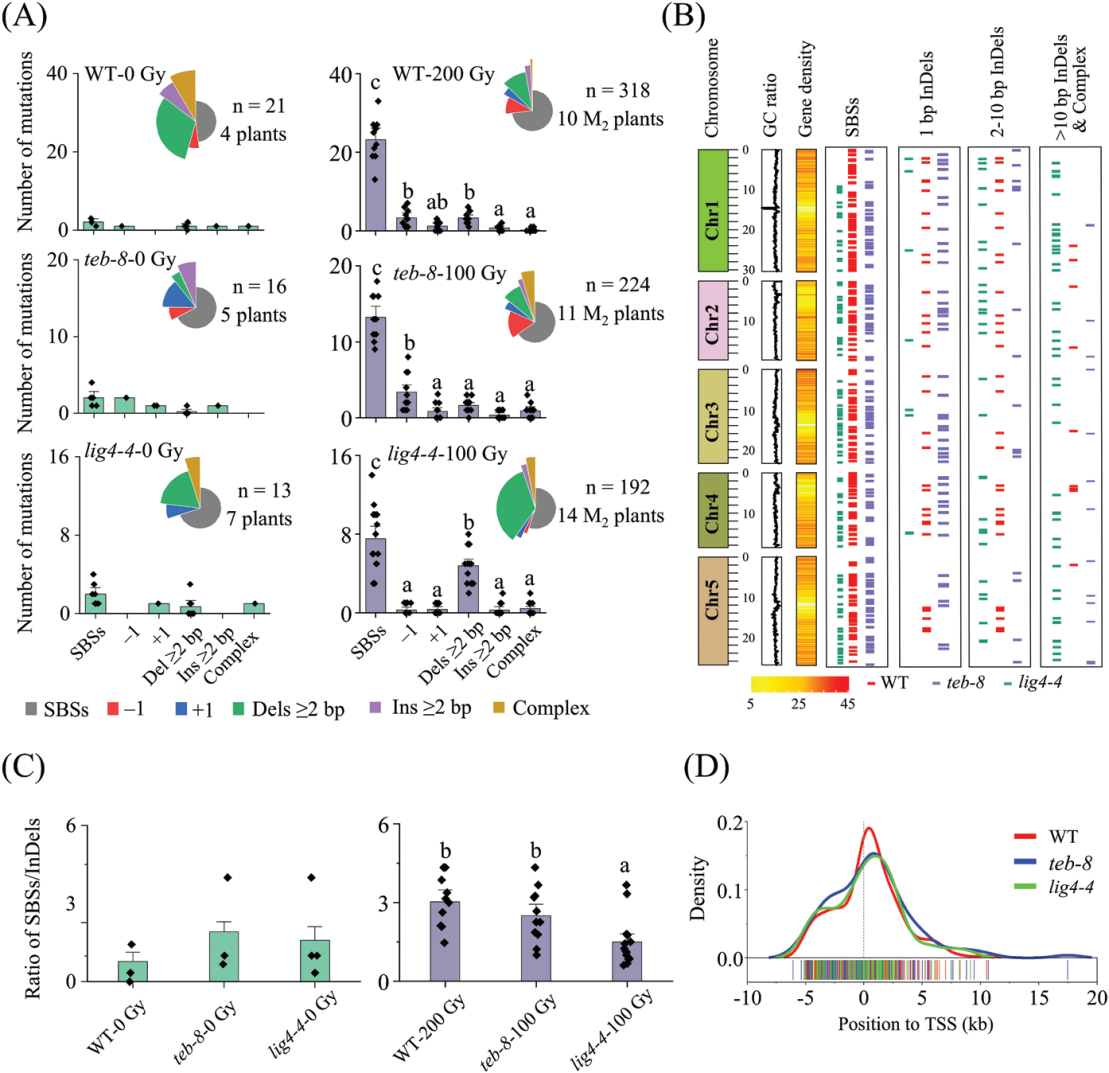
Mutations accumulated in the M_2_ generation of WT, *teb-8*, and *lig4-4* mutants under CIB irradiation. (A) Mutation types and quantity in the three lines with and without CIB irradiation exposure. Data are expressed as mean ± SEM. The solid black rhombus is the value of each individual plant. Different letters indicate significant differences among different mutation types within each line. (B) Intuitive display of SBSs and small InDels distribution across genome. (C) Ratio of SBSs to InDels in the three lines. Data are expressed as mean ± SEM. The solid black rhombus is the value of each individual plant. (D) Distribution of mutations along the TSS. Different letters indicate significant differences among the three lines (*P *< 0.05, One-way ANOVA with Duncan’s method).

In the non-irradiated group, 13 mutations were detected in the four WT plants, 16 mutations in the five *teb-8* plants, and 21 mutations in the seven *lig4-4* plants, whereas 318, 224, and 192 mutations were detected in the three genotypes irradiated with CIB (Fig. 5A and Tables S7–S8). Considering the synchronization of the genetic generation (self-bred for one generation) of the control and irradiated seeds, we conclude that the vast majority of mutations detected in the treated group were caused by CIB. Because of the small number (three for each plant) of mutations in the non-irradiated group, it was difficult to reveal their exact regularity; therefore, in the subsequent analysis, we did not consider these background mutations. Since the number of sequenced lines varied, we calculated the average mutation count or proportion for each plant.

The mutation types were distributed differently in *teb-8*, *lig4-4* and WT (Fig. 5A and B). In WT, the proportion of each type was as follows: SBSs (72.96%) > ‘−1 bp’ (10.38%) = ‘Dels ≥2 bp’ (10.06%) ≥ ‘+1’ (3.77%) ≥ ‘Ins ≥2 bp’ (2.2%) ≥ ‘complex’ (0.63%). In *teb-8*, SBSs also exhibited the highest percentage (65.18%), followed by ‘−1 bp’ (16.52%), and with no significant difference among the remaining mutation types. In *lig4-4*, SBSs also exhibited the highest percentage (55.21%), followed by ‘Dels ≥2 bp’ (34.90%), and with no significant difference among the remaining mutation types. The proportions of ‘−1 bp’ was significantly higher in *teb-8* than those in both WT and *lig4-4* (Fig. 5A and Fig. S6A). In *lig4-4,* the proportion of SBS mutations (55.21%) was significantly reduced compared to WT and *teb-8*, while the proportion of ‘Dels ≥2 bp’ (34.90%) markedly increased, reaching approximately 3.5 and 4.3 times that of WT and *teb-8*, respectively (Fig. 5A and Fig.S6A). The distribution of specific mutation types varies among the three lines were intuitive displayed in Fig. 5B. The ratio of SBSs to InDels in *lig4-4* was significantly lower than that of WT and *teb-8* (Fig. 5C). Characterizing the frequency of genome variations in terms of mutations/bp/Gy (Table 1), the mutation frequency of ‘−1 bp’ significantly increased in *teb-8*, was 2.03 and 11.75-fold that of WT and *lig4-4*, respectively; additionally, the frequency of complex mutations in *teb-8* was 8.44 folds that of WT. In *lig4-4*, the mutation frequency of SBSs was significantly lower than that of WT and *teb-8*; the mutation frequency of ‘Dels ≥2 bp’ significantly increased was approximately 2.9-fold that of WT and *teb-8* (Table 1). We also investigated the distribution of all non-intergenic variations relative to the transcription start site (TSS). The results showed that the genome mutations in the WT, *teb-8* and *lig4-4* were primarily concentrated near the TSS and decreased toward both ends (Fig. 5D). These results suggested that functional inactivation of POLQ, resulted in a significant increase in the rate of single-base deletions and complex mutations in the M_2_ genome, whereas mutation of LIG4, involved in C-NHEJ, led to a higher frequency of ‘Dels ≥2 bp’ mutation events, accompanied by a decrease in the frequency of SBSs. Overall, *lig4-4* exhibited more substantial changes in the genome variation spectrum than *teb-8*.

**Table 1.** Mutation frequency (× 10^-10^/bp/Gy)

Treatment	SBSs	–1	+1	Dels ≥ 2 bp	Ins ≥ 2 bp	Complex	Total
WT-200 Gy	9.78^b^	1.39^b^	0.51	1.35^a^	0.3	0.09^a^	13.345
*teb-8*-100 Gy	11.14^b^	2.82^c^	0.69	1.37^a^	0.31	0.76^b^	17.09
*lig4-4*-100 Gy	6.36^a^	0.24^a^	0.3	4.02^b^	0.24	0.36^ab^	11.51

Different letters indicate a significant difference among different genotype (*P <* 0.05, One-way ANOVA with Duncan’ s method).

Mutation frequency is calculated as the number of mutation events divided by the length of the reference genome, and then divided by the dose.

#### 3.4.2. The loss of TEB and LIG4 caused a pronounced impact on the spectrum of CIB-induced InDels

**Fig. 6.**
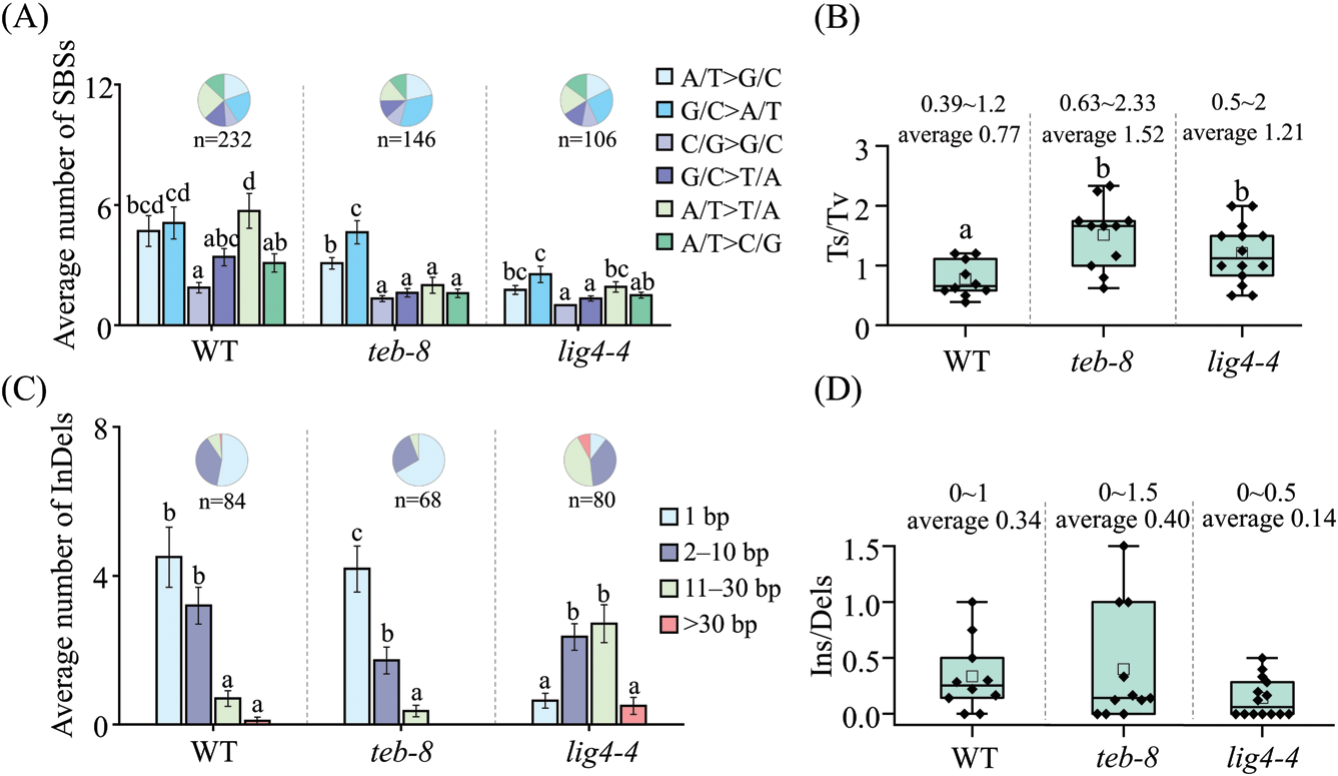
Characteristics of SBSs, InDels (A) Frequency and spectra of SBSs. (B) Ts/Tv ratio. (C) Length distribution of InDels. (D) Ins/Dels ratio. Bar chart of (A) and (C): Data are presented as the means ± SEM. Different letters indicate significant differences among various SBSs and InDels types within each line, respectively. Box plot in (B) and (D): The solid black circles represent the values of each sample; Hollow squares represent mean values; 25th–75th percentile; whiskers, 1.5 × IQR. Different letters indicate significant differences among the three lines (*P* < 0.05, One-way ANOVA with Duncan’s method).

A detailed analysis of SBSs and InDels was performed to further compare the mutation characteristics of WT, *teb-8* and *lig4-4*. SBSs can be categorized into two types: transitions (Ts) and transversions (Tv). The G/C > A/T type occupied the highest proportion in *teb-8*, whereas in the WT and *lig4-4*, A/T > G/C, G/C > A/T, and A/T > T/A had similar proportions (Fig. 6A). In the WT, the most common Tv was A/T > T/A, with an average of 5.7 per plant. In contrast, the average numbers of the four types of Tv did not differ significantly between *teb-8* and *lig4-4* (Fig. 6A). In addition, both TEB and LIG4 deficiencies resulted in a higher ratio of Ts to Tv (Ts/Tv) than that seen with the WT (Fig. 6B).

We further categorized the InDels according to their fragment lengths: 1 bp, 2 − 10 bp, 11 − 30 bp, and > 30 bp. In both the WT and *teb-8*, the number of InDels decreased as the fragment length increased. Single-base InDels were the most prevalent type, particularly *teb-8*, which comprised 67.65% of the total InDels, significantly exceeding those in *lig4-4* (11.25%) and the WT (52.94%). In *lig4-4*, the proportion of ≥ 2 bp InDels rose dramatically to 88.75%, especially the 11-30 bp InDels, its proportion showed a significant increase when compared to both WT and *teb-8* (Fig. 6C and Fig. S6B).Notably, no InDels longer than 30 bp were detected in *teb-8* (Fig. 6C). Furthermore, LIG4 deficiency reduced the Ins/Dels ratio to 0.14, which was lower than the values observed in WT (0.34) and the *teb-8* mutant (0.40). However, no statistically significant differences were detected among these groups (Fig. 6D). These results suggest that CIB irradiation is more likely to induce relatively long InDel mutations in the absence of C-NHEJ, whereas the occurrence of longer InDels decreases when Alt-EJ is blocked. This may indicate that the POLQ-mediated Alt-EJ pathway is more prone to generating longer fragment InDels under CIB irradiation.

#### 3.4.3 The loss of LIG4 caused a greater impact on microhomology dependency than the loss of TEB

**Fig. 7.**
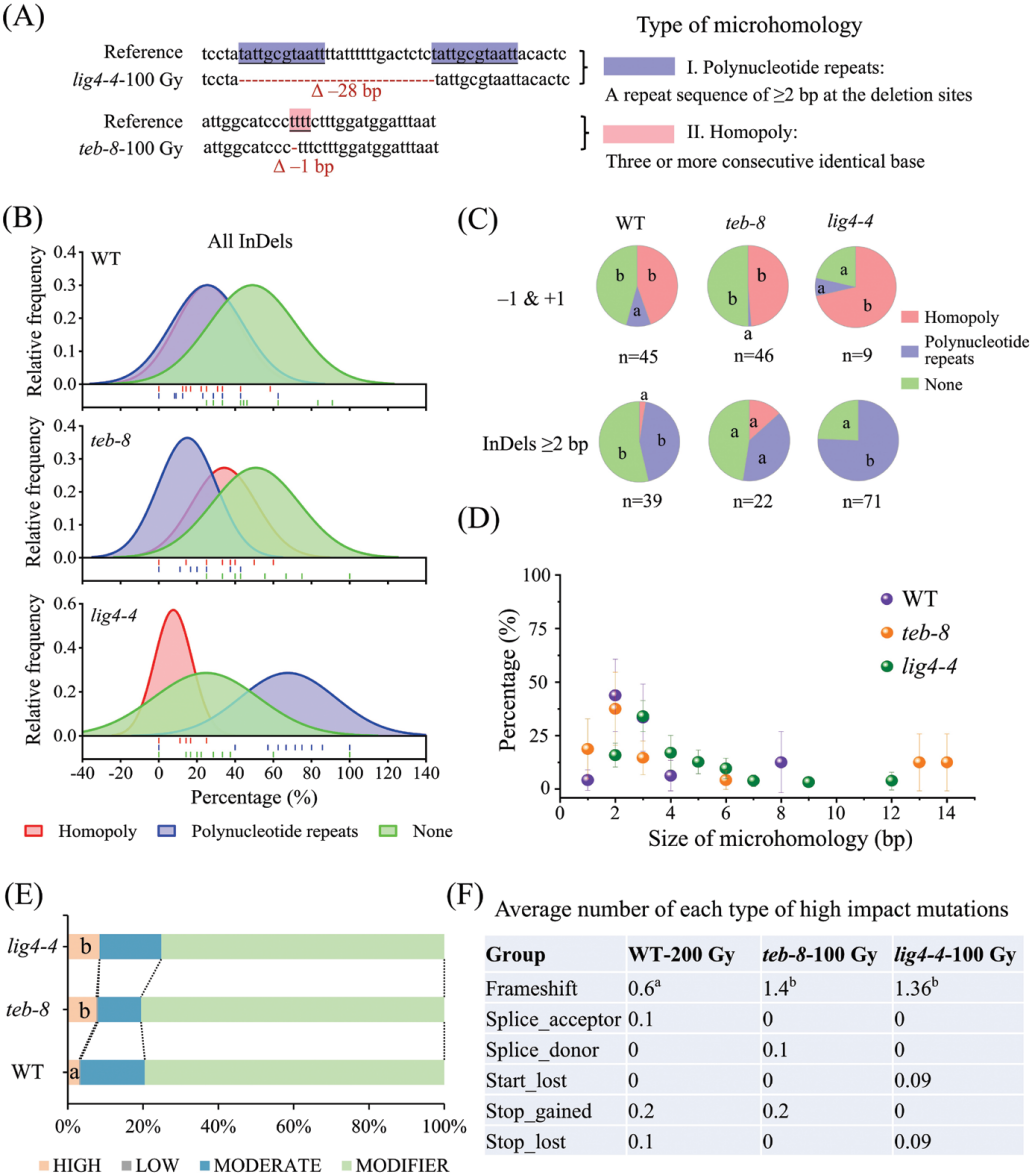
Flanking microhomology analysis of InDels. (A) Two features of microhomology sequences. (B) Distribution of the microhomology-dependency in each line. A normal distribution curve fitting approach was implemented by using the ‘Distribution + Rug’ function in OriginPro2022, lines of the rug plots (bottom) indicate the raw data (the proportion of microhomology dependency) for the individuals in each line, the vertical axis quantifying the relative frequency of data points within each partitioned interval relative to the complete dataset. (C) Microhomology dependence of single-base InDels and ‘InDels ≥2 bp’. Different letters indicate significant differences among various flanking sequence signatures within each line. (D) Distribution of the microhomology length at the rejoined site of InDels ≥ 1 bp. (E) Classification and proportional analysis of mutation impacts. (F) Classification and statistical analysis of HIGH-impact mutations. Different letters indicate significant differences among three lines (*P *< 0.05, one-way ANOVA with Duncan’s method).

The differences in InDel profiles among the WT, *teb-8* and *lig4-4* suggest a shift in the mechanism used to repair DSBs. Microhomology plays an important role in the generation of InDels via the C-NHEJ and Alt-EJ repair pathways^38^; therefore, we searched for microhomology around the flanking sequences of all InDels (Fig. 7A). *lig4-4* exhibited the highest dependence on polynucleotide repeats (67.62%) and the lowest homopoly dependence (7.60%) than WT and *teb-8*, leaving only 24.78% independent of microhomology (Fig. 7B and Fig. S6C). In contrast, only approximately 50% of InDels in both WT and *teb-8* were classified as microhomology-mediated events (Fig. 7B and Fig. S6C). The dependence of ‘InDels ≥2 bp’ on polynucleotide repeats was much higher than that of single-base InDels (Fig. 7C). For example, there were 43.83%, 39.17% and 75.63% of ‘InDels ≥2 bp’ depended on polynucleotide repeats in WT, *teb-8* and *lig4-4*, respectively, whereas only 9.78%, 1.14% and 7.14% of single-base InDels depended on polynucleotide repeats. Further analysis of the microhomology lengths in ‘InDels ≥2 bp’ revealed that compared to WT, which was distributed at 1–4 bp and 8 bp with a peak at 2 bp, *teb-8* and *lig4-4* exhibited a wider distribution (1–14 bp), with microhomology peaks at 2 bp and 3 bp, respectively (Fig. 7D).

#### 3.4.4. Loss of function in LIG4 and TEB induced a higher proportion of high-impact genome mutations than WT even at lower doses

The putative impact of the variant was categorized into four categories–HIGH, MODERATE, LOW, and MODIFIER–according to its effects on genes and proteins using SnpEff (Fig. 7E and Table S8). HIGH-impact mutations include several subclasses, such as frameshift, start/stop gain/lost, splice donor, and acceptor variants (Fig. 7F). MODERATE impact primarily includes missense and 5′/3′ untranslated region variant, whereas LOW impact covers synonymous and intron mutations. Mutations occurred in the intergenic, upstream, and downstream regions of the MODIFIER impact. We calculated the average proportions of the above four categories in each re-sequenced line and found that the proportion of HIGH-impact mutations was significantly higher in *teb-8* and *lig4-4* than in WT, corresponding to 2.5 and 2.8 folds that of the WT, respectively (Fig. 7E). Further analysis revealed that the average number of frameshift mutations per plant was significantly higher in *teb-8* and *lig4-4* mutants than in the WT even when the irradiation dose was reduced by half. These findings suggest that the loss of function in LIG4 and TEB results in a higher proportion of HIGH-impact genomic mutations than in the WT, which are more likely to alter gene and protein functions.

#### 3.4.5. Higher frequencies of SVs occur in *lig4-4*

To investigate the influence of different DNA repair pathways on genomic SVs induced by CIB, we performed SVs detection in the irradiated WT, *teb-8* and *lig4-4*, using three established algorithms—Lumpy, Delly, and Manta—followed by manual validation via IGV (Fig. S7). Our analysis revealed striking differences in SVs profiles across genotypes (Table 2). In *lig4-4* mutants (*n* = 14), we identified 18 SVs, predominantly large deletions (17 out of 18), with one duplication event. The deletion sizes ranged from 55 bp to 657 bp. In contrast, WT plants (*n* = 10) exhibited only two deletions, one of which was remarkably large (18 kb). Notably, no SVs were detected in *teb-8*, consistent with prior InDels analyses using VarScan2, further supporting the absence of large deletion events in this genotype. These findings suggest that loss of LIG4 function disrupts C-NHEJ, shifting DNA repair toward error-prone pathways that generate extensive genomic rearrangements. Conversely, intact C-NHEJ in *teb-8* maintains genomic stability by avoiding a large accumulation of SVs.

**Table 2.** Genomic SVs detected by Lumpy, Delly, and Manta

Group	Sample	Chromosome	Start position	End position	SVs type	SVs size (bp)	IGV verification
*lig4-4*-100 Gy	L1-2	1	6992317	6992386	Deletion	69	✓
*lig4-4*-100 Gy	L1-4	1	10993642	10993984	Deletion	342	✓
*lig4-4*-100 Gy	L1-11	1	16939998	16940064	Deletion	66	✓
*lig4-4*-100 Gy	L1-12	1	25618829	25618891	Deletion	62	✓
*lig4-4*-100 Gy	L1-14	1	27307872	27308242	Deletion	370	✓
*lig4-4*-100 Gy	L1-4	1	28491172	28491229	Deletion	57	✓
*lig4-4*-100 Gy	L1-2	1	30164546	30165031	Deletion	485	✓
*lig4-4*-100 Gy	L1-12	2	10395128	10395225	Duplication	97	✓
*lig4-4*-100 Gy	L1-15	3	19928674	19928724	Deletion	50	✓
*lig4-4*-100 Gy	L1-11	3	21916848	21916914	Deletion	66	✓
*lig4-4*-100 Gy	L1-8	4	195851	195942	Deletion	86	✓
*lig4-4*-100 Gy	L1-12	4	16040504	16040567	Deletion	63	✓
*lig4-4*-100 Gy	L1-7	4	18179116	18179773	Deletion	657	✓
*lig4-4*-100 Gy	L1-6	4	18289363	18289512	Deletion	149	✓
*lig4-4*-100 Gy	L1-9	5	1745827	1745882	Deletion	55	✓
*lig4-4*-100 Gy	L1-13	5	5531895	5531960	Deletion	65	✓
*lig4-4*-100 Gy	L1-7	5	17879971	17880025	Deletion	54	✓
*lig4-4*-100 Gy	L1-7	5	20646146	20646201	Deletion	55	✓
WT-200 Gy	3-4	1	21677471	21677532	Deletion	61	✓
WT-200 Gy	3-1	3	307505	326093	Deletion	18588	✓

Sample refers to the individual plants used for sequencing.

## 4. Discussion

### 4.1. Combination of C-NHEJ deficiency and High-LET radiation is an effective strategy to highlight the role of Alt-EJ in higher plant

Genomic variations caused by erroneous or imprecise DNA repair are important sources of genetic diversity. DSBs repair via C-NHEJ and Alt-EJ was more error-prone than HR repair. From the perspective of genetic variation creation, a combination of these two pathways and CIB provides a novel strategy for repair-driven heavy-ion beam mutagenesis. Alt-EJ plays a role in DSBs repair and is inhibited by C-NHEJ and HR in higher eukaryotes. However, the exact consequences of the Alt-EJ remain unclear. In the field of plant research, few studies have focused on the role of Alt-EJ in repairing complex DNA damage induced by CIB, and little attention has been paid to the impact of Alt-EJ on the genome mutation spectrum in the M_2_ generation, although this is important for mutation breeding using heavy-ion beam irradiation. Toward this end, here, we considered the following two aspects. First, high-LET ionizing radiation (CIB), which causes clustered DSBs, was selected because previous studies have reported that it reduces the dependence of DSBs repair on C-NHEJ and enhances the utilization of Alt-EJ by promoting DNA end-resection.^39^ Second, a specific genetic background (C-NHEJ-deficient) was employed to further enhance the mutagenic effects of heavy-ion beam radiation mediated by Alt-EJ. POLQ and LIG4 are the key factors in Alt-EJ and C-NHEJ, respectively. Here, documented null mutation of TEB and LIG4 T-DNA insertion lines (*teb-8* and *lig4-4*) were used to investigate the involvement of Alt-EJ in CIB-induced genome instability.^40,41^

By conjoint analysis of *teb-8* and *lig4-4*, we can indirectly study the independent contributions of Alt-EJ to the genomic mutation spectrum. In WT, the outcomes of repair represent the collective contributions of all active pathways, even C-NHEJ and HR are widely regarded as the predominant mechanism, but Alt-EJ is normal to exercise its function.

### 4.2. Different allelic mutants of POLQ showed varying sensitivity to CIB radiation because of the T-DNA insertion sites, but both less sensitivity than *lig4*

It remains controversial whether the *polq* mutant displays developmental defects under normal growth conditions.^15,18,42^ The two *teb* allele mutants (*teb-3* and *teb-8*) used in this study did not display deformed leaves as *teb-2* and *teb-5* which reported in previous studies,^15,42^ except for slightly shorter roots than those of the WT. Rice *polq* mutants (with insertion sites analogous to the *A. thaliana teb-2* allele) exhibited normal developmental phenotypes under standard growth conditions.^43^ POLQ encodes an A-family polymerase containing three functionally distinct domains: an N—terminal helicase domain that can counteract RPA and RAD51, thereby hindering HR, a C-terminal polymerase domain for DNA synthesis, and a flexible central region that may be responsible for substrate selection of POLQ.^44^ In this study, the roots of *teb-8* were slightly shorter than those of *teb-3* (Fig. 1C–D). A previous study reported that the roots length of *teb-1*, which contains a 2.7 kb deletion in the helicase domain, was approximately one-third that of the WT; however, this severely stunted growth was not observed in other allele mutants.^15^ The T-DNA insertion site in *teb-8* is located in the 14th intron in the central region near the helicase domain, whereas the insertion site is located in the polymerase domain in *teb-3*. In summary, it appears that the phenotype of *teb* mutants is highly variable under different growth conditions and across plant species and that T-DNA insertions located at different positions in the POLQ have distinct effects on the development of various allele mutants.

Although previous studies have demonstrated that *lig4* exhibits significantly higher sensitivity to genotoxic stresses inducing DSBs than WT, it remains controversial whether the absence of POLQ displays a similar feature and how its sensitivity compares to that of *lig4*. Studies across multiple species have shown that the absence of POLQ results in hypersensitivity to bleomycin.^20,45,46^ Sensitivity of *Physcomitrella patens* to bleomycin followed the order: *polq* < WT < *lig4*. This result was interpreted as cells deficient in POLQ repairing a large number of DSBs via more accurate C-NHEJ or HR pathways, thereby exhibiting stronger resistance to bleomycin.^18^ However, this finding is in strong contrast to data reported for higher plants. For instance, in rice, the *com1-1* (involved in Alt-EJ) mutant exhibited lower sensitivity to bleomycin than the *lig4* mutant but showed greater sensitivity than the WT.^47^ In our study, both *teb* and *lig4* mutants exhibited higher sensitivity to CIB or zeocin than the WT, and *lig4-4* was more sensitive to these genotoxic treatments than *teb-8* and *teb-3*. Among the different POLQ allelic mutants, the sensitivity of *teb-8* to CIB and zeocin was greater than that of *teb-3*. Our findings on radiation sensitivity to CIB differ from those of a recently published study, which reported that under CIB treatment, *teb-5* exhibited greater radiation sensitivity than *lig4*.^48^ This discrepancy may be attributed to variations in irradiation parameters and the specific *teb* mutants used. Compared to the DNA polymerase domain, the helicase domain and central region of POLQ may play a more important role in responding to DSBs. For example, *teb-1* is more sensitive to methyl methanesulfonate and mitomycin C than *teb-3*.^15^ The helicase activity of POLQ is crucial for protecting plants from endogenous and exogenous NO toxicity.^40^ Overall, the radiation sensitivity of *lig4* mutants was higher than that of POLQ mutants, indirectly supporting the idea that in the absence of C-NHEJ, the higher error-prone repair tendency of Alt-EJ poses a serious threat to genome stability, leading to reduced tolerance to DSBs.

Unlike TEB, LIG4 has been demonstrated in a series of reports that homozygous mutants such as *lig4-1* (T-DNA insertion in the 1st exon of LIG4), *lig4-2* (possessing an insertion in a region encompassing part of the eighteenth intron and nineteenth exon of LIG4), *lig4-3* (SALK_095962, T-DNA insertion in the 9th intron of LIG4), *lig4-4* (SALK_044027, T-DNA insertion in the 6th exon of LIG4) are extremely sensitive to DSBs but remain viable, fertile, and phenotypically normal in the absence of exogenous DNA-damaging stimuli. Few studies have compared the functional differences among different T-DNA insertion mutants of LIG4, which may be attributed to its lack of well-defined functional domains—unlike the TEB gene. Most reports on *Atlig4* describe these mutants (in case of irradiation, chemical agent, gene editing, T-DNA integration) as NHEJ-defective plants.^6,41,49–52^

### 4.3. The genome mutation is jointly shaped by exogenous DNA damage induction and cellular repair status

The DDR coordinates processes such as DNA repair, replication, cell cycle regulation, cell death, and transcriptional regulation. Together, these processes maintain normal cell function, growth, development, and genomic stability. Transcriptome analysis revealed that the absence of *teb* and *lig4* resulted in markedly distinct DDR patterns. For instance, the *teb-8* mutant exhibits constitutive DDR, whereas DDR in the *lig4-4* mutant is only activated following CIB exposure. Previous studies have reported that endogenous DSBs always occur during normal DNA replication in the cell cycle.^53^ Considering the presence of constitutive DDR and endogenous DSBs in *teb-8* mutants, as well as the error-prone nature of C-NHEJ, it is reasonable to predict that genomic variation in POLQ mutants would occur more frequently than in the WT or *lig4* in the non-irradiated group. However, our resequencing data showed that the average number of mutations in the control groups of WT, *teb-8* and *lig4-4* was approximately three. In order to ascertain whether there is a possibility that some of the changes observed in the mutants (phenotypes and sensitivity to CIB) are caused by mutations unrelated to the presumed disruption of the DNA repair system in *teb* or *lig4*, we annotated the detected mutations in unirradiated groups of these two lines by SnpEff (Table S7). We found that no DDR-related genes were directly identified. Combined with the phenotypic observations of these mutants under nature condition (without irradiation treatment), we infer that the sensitivity of mutant lines to CIB is primarily caused by mutations in the *TEB* or *LIG4* genes. When treated with CIB irradiation, the number of genomic mutations increased significantly, and the spectrum varied by genotype. These findings confirmed that genomic mutations were jointly shaped by exogenous DNA damage induction and cellular repair status.

Additionally, our results showed that mutations were primarily concentrated within a 10 kb range on either side of the TSS. Possible explanations for this phenomenon are as follows: (i) Previous studies have shown that the number of inverted repeats (IRs) in the genome gradually decreases along both sides of the TSS.^54^ Therefore, IR sequences may be key factors in heavy-ion beam induced heritable mutations. (ii) The open chromatin structure and higher accessibility around TSS. For instance, by using the ATAC-seq technique, Grandi demonstrated that chromatin is usually in an open state around TSS.^55^ This characteristic facilitates the accessibility of repair factors, which also include error-prone repair.^24^ (iii) Transcription processes may be involved in DSBs formation near TSS, which in turn may affect genomic stability at this region. The region of TSS may accumulate R-loops that caused by abnormal transcription elongation under stress or DNA damage.^24,56^ R-loops increase DNA complexity, and interfere with DNA replication and repair processes, this might be one of the possible reasons leading to increased mutation rates in TSS-proximal regions. It has been reported that DSBs and translocations are enriched around active TSSs in neural stem/progenitor cells (NSPCs).^57^

### 4.4. Manipulating DNA repair pathways effectively adjusts the InDels profile and enhances the mutation impacts on genes induced by CIB

Alt-EJ was initially regarded as a backup repair mechanism upon its discovery. However, subsequent investigations have revealed that Alt-EJ performs essential biological functions even when both HR and C-NHEJ pathways remain intact.^38^ It competes with HR/C-NHEJ, evidenced by POLQ-mediated HR suppression via RAD51 binding and nucleofilament inhibition. In G1-phase cells, where HR is unavailable and resection impedes KU protein binding, Alt-EJ becomes predominant.^27^ For high-LET CIB-induced complex DSBs, Alt-EJ competes with HR while exhibiting unique advantages in complex breaks processing.^25^ In the present study, the genome mutation profiles of WT encompass repair signatures of both Alt-EJ and C-NHEJ, demonstrating that Alt-EJ can functionally operate even when C-NHEJ and other major repair pathways remain intact. Thus, we propose that Alt-EJ serves not as a backup but as an active repair pathway in plant responses to CIB-induced DSBs.

Compared to the WT, *lig4-4* exhibited a dramatic change in the mutation spectrum, whereas *teb-8* showed a moderate change. The biggest difference between *teb-8* and *lig4-4* under CIB irradiation was observed in the spectrum of the small InDels and SVs (Fig. 6C and Table 2). This phenomenon aligns with Alt-EJ’s error-prone repair mechanism that leads to high rates of large deletions and SVs. *teb-8* showed a significant increase in single-base InDels and a reduction in ≥ 2 bp InDels. Additionally, > 30 bp InDels and SVs were not detected in *teb-8*. This is reasonable because C-NHEJ is predominated in *teb-8*. These findings further reveal the important role of Alt-EJ on CIB-induced genome mutation profile. Importantly, loss-of-function in LIG4 and TEB induced a higher proportion of high-impact genome mutations, especially frameshift mutations than in the WT, even at lower doses. Due to the technical limitations of next-generation sequencing (NGS), the detection rate of SVs remains relatively limited. Future research could focus on combining the third-generation sequencing technique with M_1_ generation on a single cell level to specifically investigate the role of Alt-EJ for the CIB-induced SVs profiles.

Alt-EJ employs microhomology for strand annealing; most of the time, end resection is required to expose the regions. Subsequent end-joining of the resected DNA in this region may lead to microhomology-flanked deletions and templated insertions.^26^ Studies in animal cells have indicated that Alt-EJ typically utilizes 2–20 bp microhomology to facilitate the repair process.^12,38,58^ In the absence of POLQ in *Physcomitrella patens*, the frequency of deletions involving microhomology (4 bp) decreases from 73% (WT) to 7.8%.^18^ Because Alt-EJ utilizes microhomology, a lower reliance on microhomology is expected in the *teb-8* mutant. In the present study, as C-NHEJ remains the major used pathways, the dependency of InDels on microhomology in *teb-8* was similar to that in WT, however, a significantly increased proportion of polynucleotide repeats was detected in the *lig4-4* mutant (Fig. 7 and Fig. S6C). Therefore, Alt-EJ could preferentially contribute to the generation of polynucleotide repeats-dependent InDels in *lig4-4* mutant.

In the case of ZFN-mediated targeted mutagenesis and GT in *A. thaliana*, approximately 90% of deletions were independent of microhomology or used only 1 bp microhomology in WT or *smc6b* (involved in HR)-deficient lines. In contrast, a microhomology > 2 bp is commonly used in C-NHEJ-deficient lines.^59^ Upon gamma-ray irradiation, the dependence on microhomology in *lig4* mutants increases, with 4 bp being the most common type.^37^ In addition, previous studies and our present results demonstrated that the size of InDels might be closely related to the type of microhomology.^30,60,61^ Single-base InDels show a stronger association with homopoly sequence than with polynucleotide repeats; InDels ≥ 2bp exhibit a significant correlation with polynucleotide repeats. Here, the ≥ 2 bp InDels in *lig4-4* mainly rely on polynucleotide repeats, and the max size of microhomology in WT, *teb-8*, and *lig4-4* was 8, 14, and 12 bp, respectively, which were longer than the case of ZFN-mediated targeted mutagenesis and GT. Under 1000 Gy gamma-ray irradiation, the microhomology in WT plants was primarily 2 bp.^37^ Under CIB irradiation, we found that the dependence on microhomology in the WT and *teb-8* was also primarily at 2 bp, whereas in *lig4-4* it was primarily at 3 bp. This suggests that disruption of C-NHEJ promotes a pathway shift toward microhomology-dependent repair mechanisms.

### 4.5. Proportion and frequency of SBSs was decreased in *lig4-4*

The proportion of SBSs in *teb-8* showed a slight decline compared to WT, although it was not statistically significant, while significantly decreased in *lig4-4* (Fig. 5A, Fig. S6A, and Table 1). POLQ is a unique DNA polymerase that can execute Alt-EJ and TLS across oxidative or AP lesions. TLS has low fidelity and is one of the reasons for genome mutation, including SBSs. The different degrees of variation in SBSs between *teb-8* vs. WT, and *lig4-4* vs. WT could be attributed to several factors. Firstly, the usage rate of TLS is very limited and it is usually activated when other repair approaches fail to handle specific types of DNA damage.^62^ Secondly, CIB induces a complex DNA damage, which may involve not only base damage that can be bypassed by POLQ but also more severe clustered DSBs. In such cases, the role of TLS may be overshadowed by the main repair pathways, such as C-NHEJ and HR. Thirdly, the generation of SBSs is not solely determined by the activity of TLS. For instance, the deficiency in MMR caused SBSs rate be above 100-fold higher than that in WT,^63^ and this indicated that MMR played important roles in generation of SBSs.

**Fig. 8.**
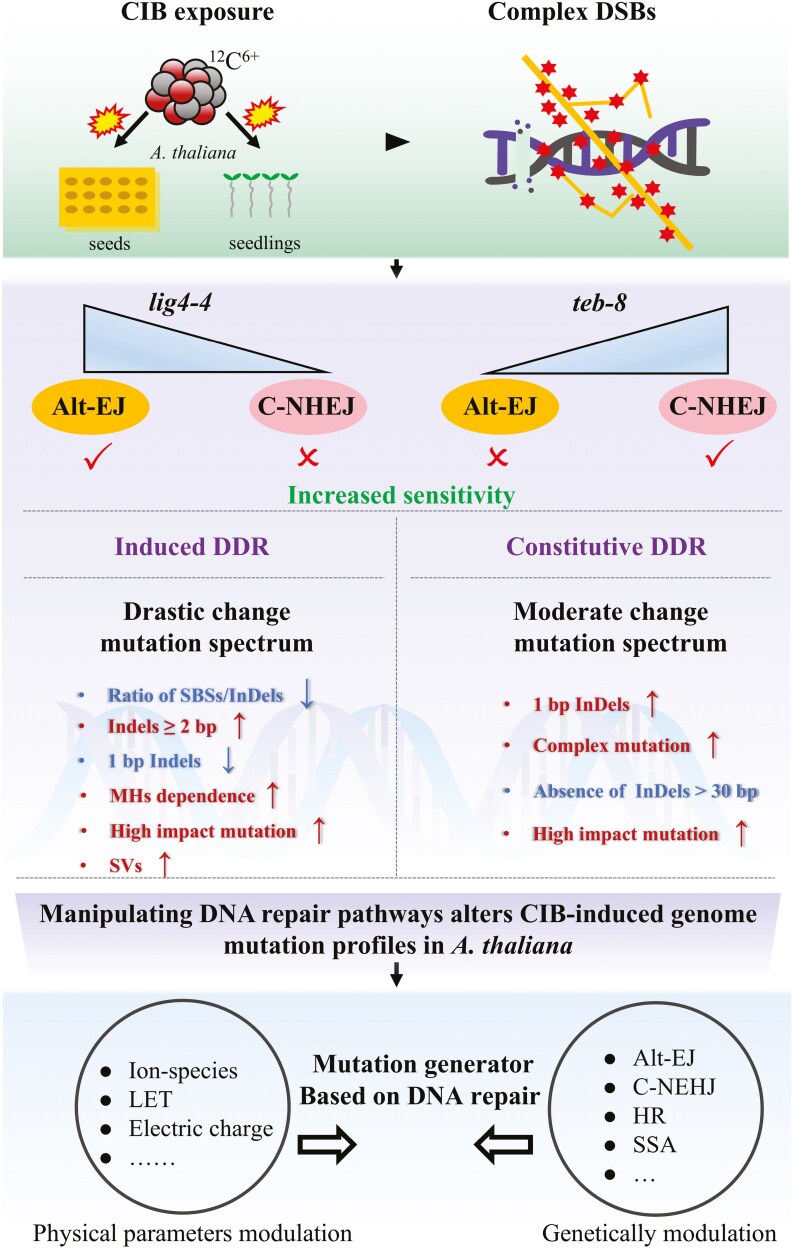
Overview of radiosensitivity, DDR, and repair consequence of *teb-8* and *lig4-4* under CIB exposure.

In summary, sensitivity and genome mutation analyses of different mutant lines revealed the critical role of Alt-EJ in DNA damage repair in *A. thaliana* exposed to CIB radiation. Modulating the Alt-EJ pathway can regulate the InDels mutation spectrum induced by CIB irradiation, and induce an enrichment effect of high-impact mutations even at lower doses (Fig. 8). Future research will focus on changing the balance among existing DNA repair pathways by (i) regulating the physical parameters (LET, ion species, and electric charge) of heavy-ion beam to induce different patterns of biological damage; (ii) targeting different factors of DSBs repair that specifically respond to ionizing radiation, especially when growth in the natural environment is not affected by the genotype. This strategy is expected to endow CIB mutagenesis with some mouldability at the genome level, while developing a more efficient mutation reactor.

## Supplementary Material

dsaf014_suppl_Supplementary_Table_S1

dsaf014_suppl_Supplementary_Table_S2

dsaf014_suppl_Supplementary_Table_S3

dsaf014_suppl_Supplementary_Table_S4

dsaf014_suppl_Supplementary_Table_S5

dsaf014_suppl_Supplementary_Table_S6

dsaf014_suppl_Supplementary_Table_S7

dsaf014_suppl_Supplementary_Table_S8

dsaf014_suppl_Supplementary_Figures_S1-S7

## Data Availability

The raw sequence data reported in this paper have been deposited in the Genome Sequence Archive in National Genomics Data Center, China National Center for Bioinformation / Beijing Institute of Genomics, Chinese Academy of Sciences (GSA, Accession: CRA019100) that are publicly accessible at https://ngdc.cncb.ac.cn/gsa.
