# Supplementary material for: Manipulating alternative end-joining alters carbon-ion beam-induced genome mutation profiles in Arabidopsis thaliana
Source: DNA Res. 2025 May 31;32(4):dsaf014. doi: 10.1093/dnares/dsaf014 (PMC12231562; doi:10.1093/dnares/dsaf014)
Supplement: dsaf014_suppl_Supplementary_Figures_S1-S7 [file dsaf014_suppl_supplementary_figures_s1-s7.docx]

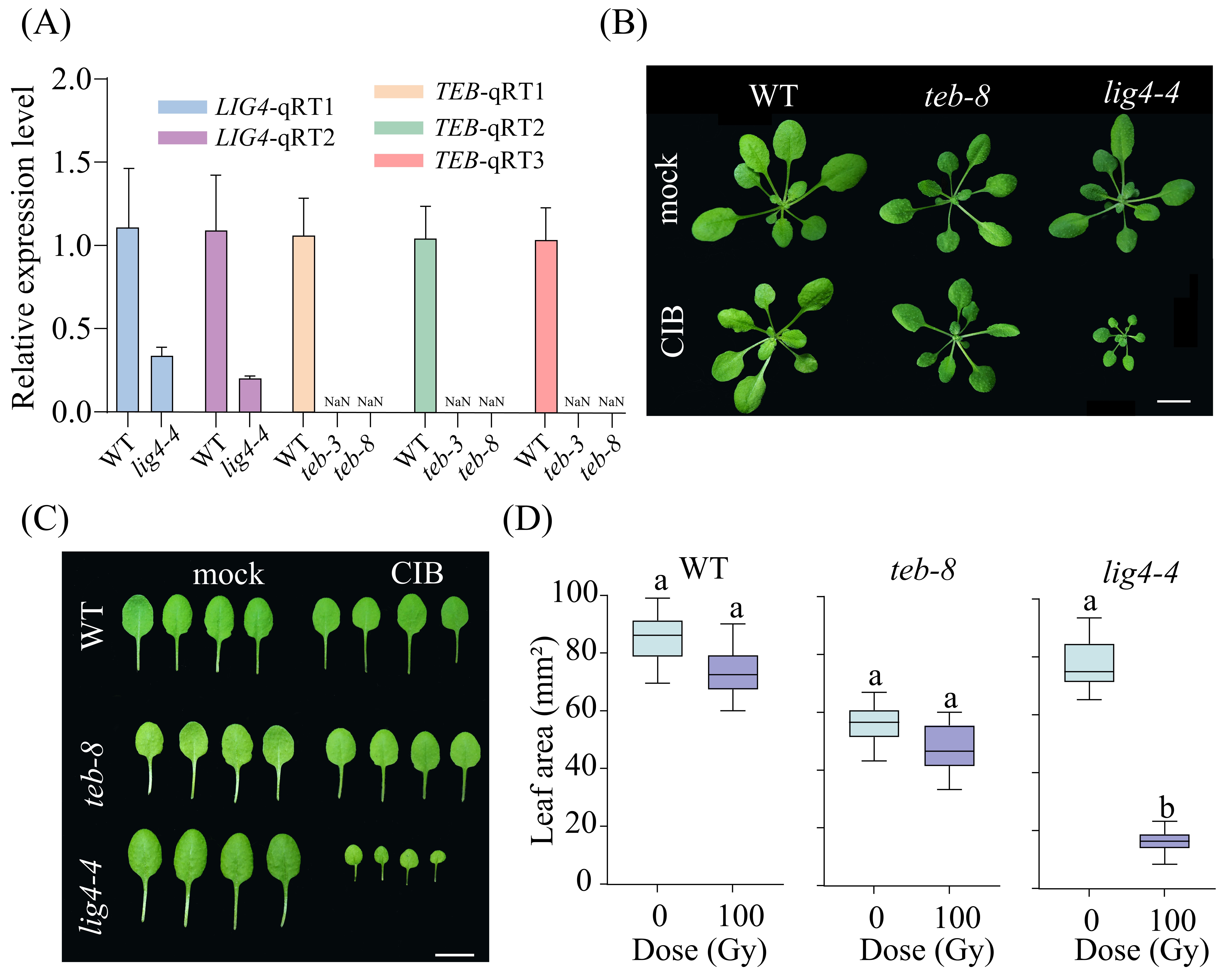


**Figure S1.** Phenotype of WT and mutants under CIB irradiation. (A) qRT-PCR quantification of *TEB* or *LIG4* gene expression in *teb* and *lig4* mutants, NaN indicates no value. The positions of the primer pairs are indicated by the corresponding arrows in (Fig. 1A), and the primer sequences are shown in Table S3. means ± SEM. (B) The representative images of WT, *teb-8*, and *lig4-4* plants grown for 24 d under CIB irradiation. Bar = 1cm. (C) Leaf phenotypes of WT and mutants with and without CIB irradiation at a dose of 100 Gy, grown in soil for 24 d. Bar = 1cm. (D) Rosette leaf area of WT, *teb-8*, and *lig4-4* mutants after 24 days of growth under standard conditions. Different letters indicate significant differences (n = 27-35, means ± SEM, Student's *t*-test, *P* < 0.05).


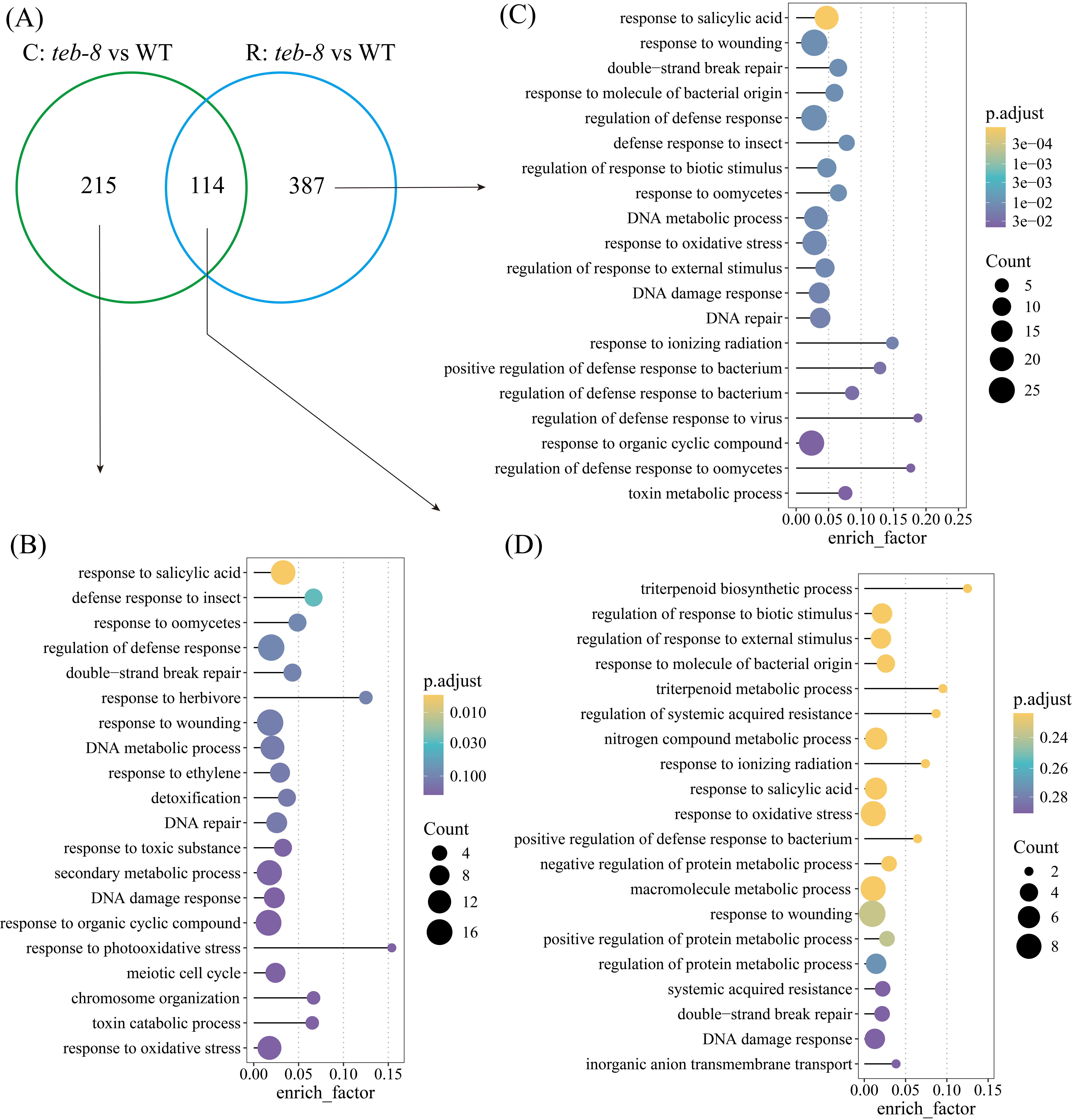


**Figure S2.** Enrichment of DEGs in *teb-8* vs WT under irradiated and non-irradiated conditions. (A) A Venn diagram showing the number of DEGs in *teb-8* versus WT under non-irradiated “C” and irradiated “R” conditions. (B) GO analysis of the 215 unique DEGs in C: *teb-8* vs WT. (C) GO analysis of the 387 unique DEGs in R: *teb-8* vs WT. (D) GO analysis of the 114 shared DEGs in both C: *teb-8* vs WT and R: *teb-8* vs WT. The size and color of the circles indicate the rich factor and significance value (p. adjust), respectively.


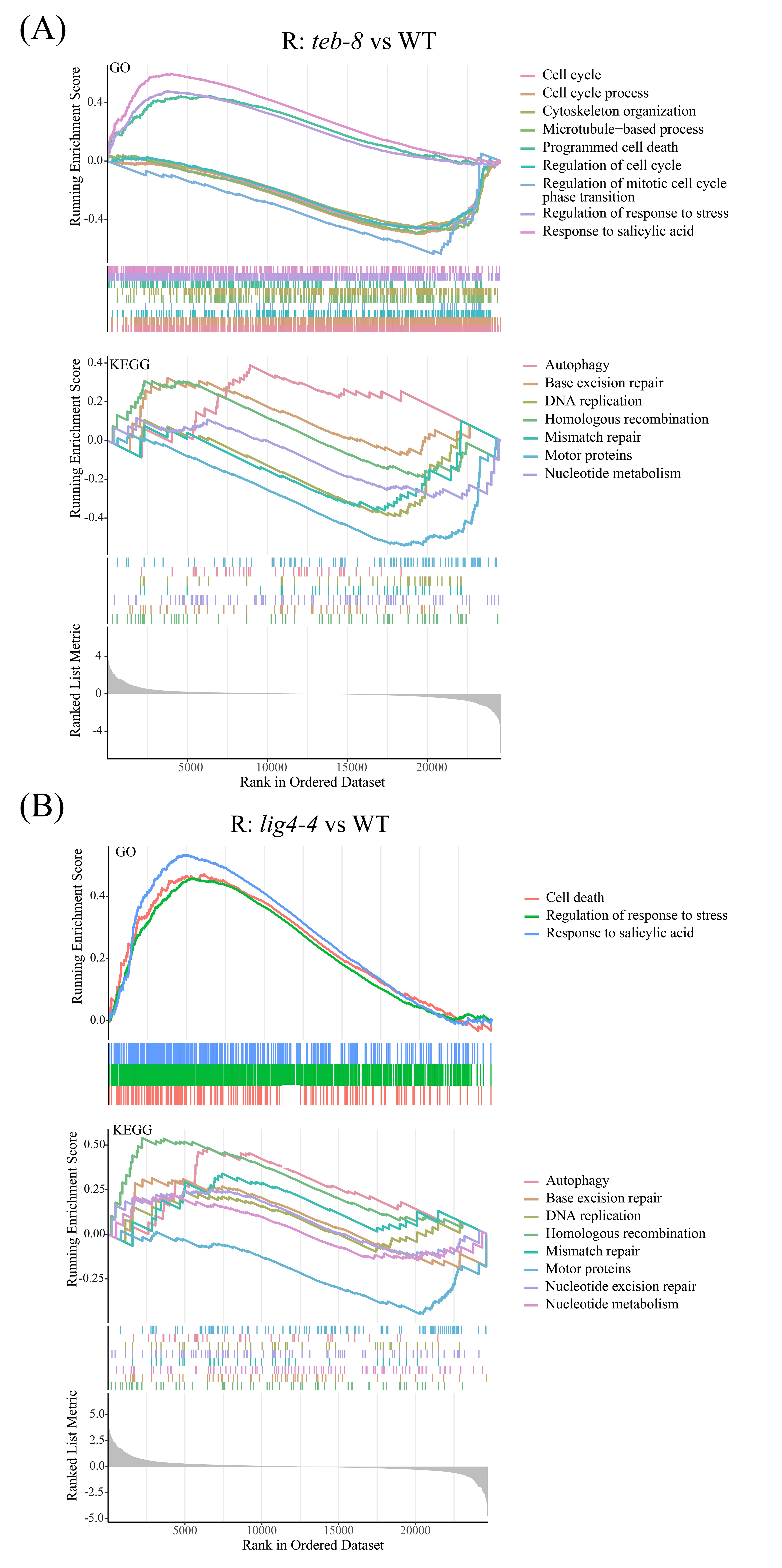


**Figure S3.** GSEA enrichment analysis. (A) GSEA enrichment analysis of the R: *teb-8* vs WT comparison group; (B) GSEA enrichment analysis of the R: *lig4-4* vs WT comparison group. The positive enrichment score indicates upregulation of the pathway, while the negative score indicates downregulation.


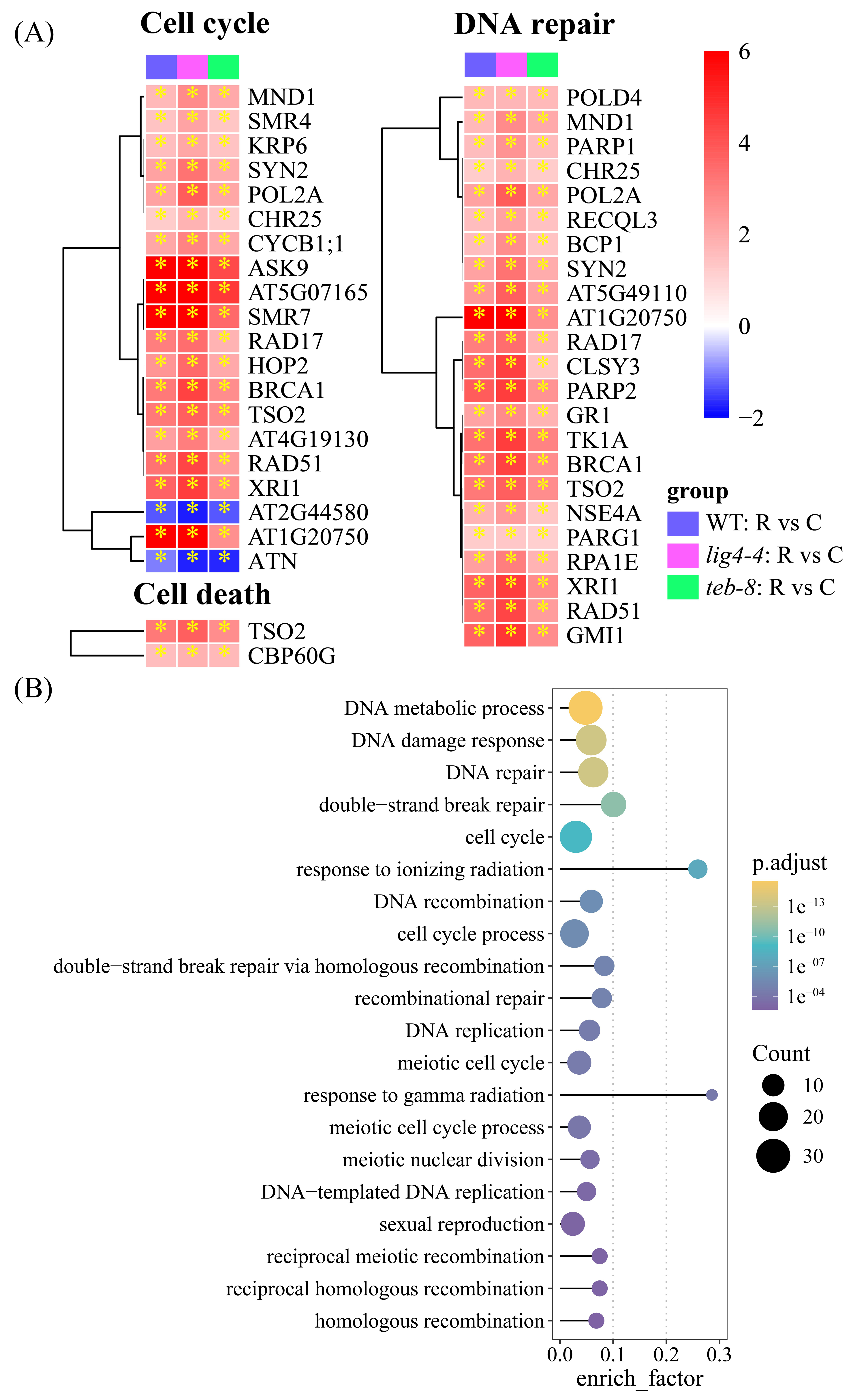


**Figure S4.** Analysis of DEGs common to the CIB-irradiated (“R”) and non-irradiated (“C”) groups of the *teb-8*, *lig4-4*, and WT lines. (A) Transcription-level changes of the 33 DDR-related DEGs. The heatmap was created using log_2_ (fold change), with red representing up-regulated genes and blue representing down-regulated genes. (B) GO (biological processes only) enrichment of 167 DEGs in three compare groups. The size and color of the circles indicate the rich factor and significance value (p. adjust), respectively.


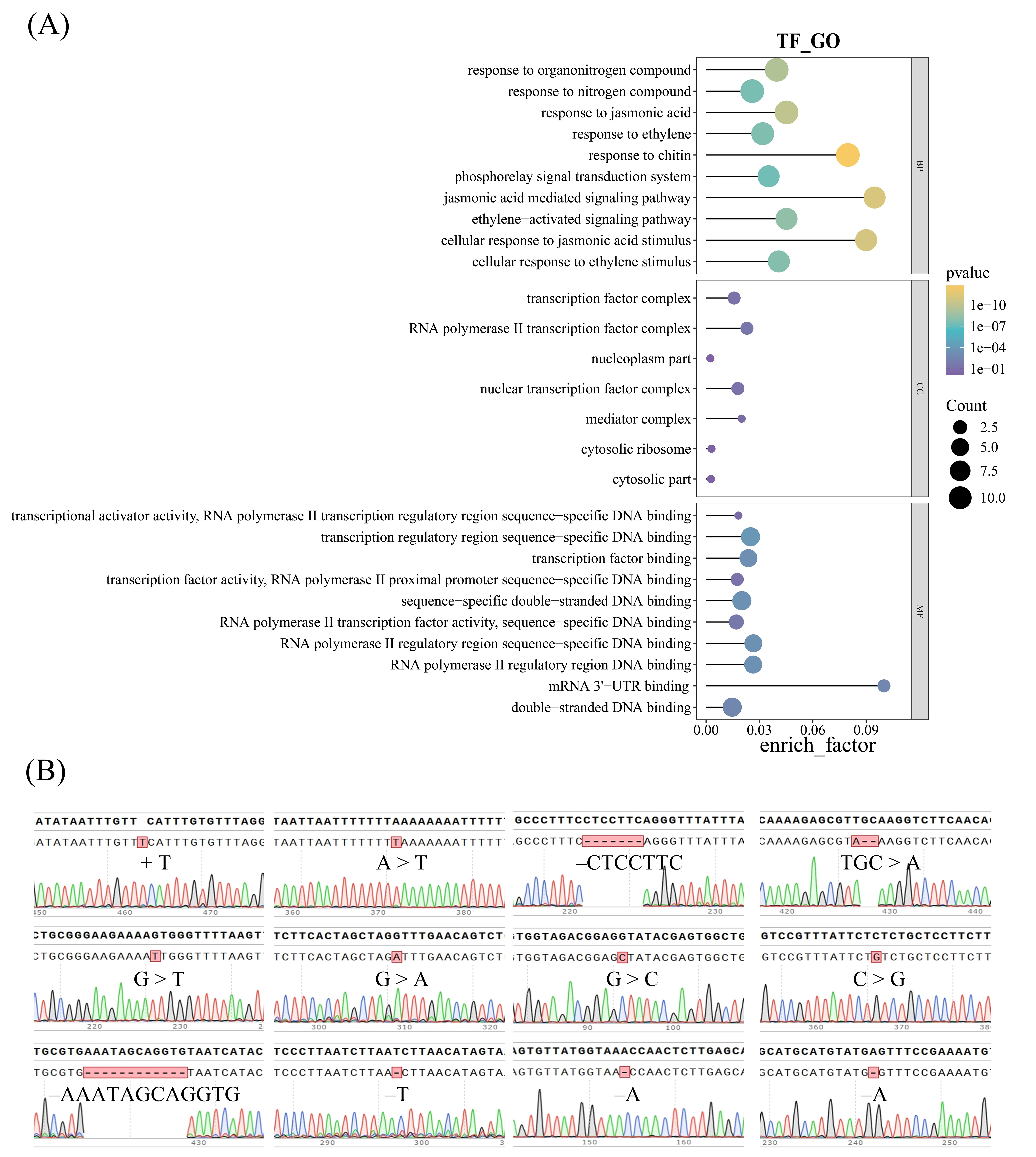


**Figure S5.** GO enrichment of TFs and sanger sequencing validation. (A) GO enrichment of 68 TFs. The size and color of the circles indicate the rich factor and significance value (p.value), respectively. (B) Sanger sequencing of mutation sites detected by whole genome resequencing.


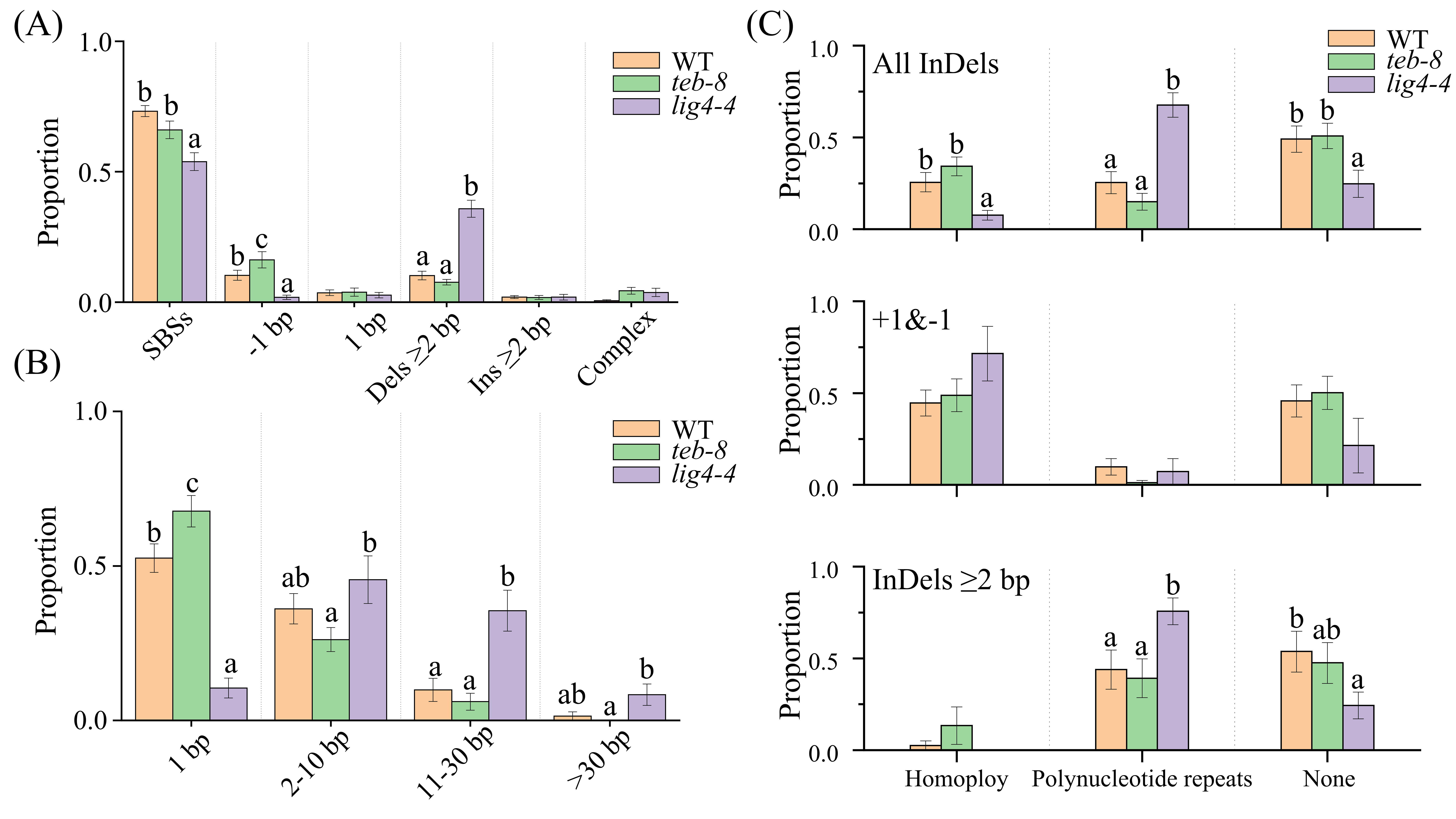


**Figure S6.** Significance supplementation analysis of genomic data. (A) Significance analysis of the pie chart showing the proportion of mutations under irradiation conditions in Fig. 5A. (B) Significance analysis of the pie chart presenting the proportion of InDels in three lines in Fig. 6C. (C) Significance analysis of the proportion of flanking microhomology sequences of InDels in three lines in Fig. 7A and C. Different letters indicate significant differences among the three lines (*P* < 0.05, means ± SEM, One-way ANOVA with Duncan’s method).

| Information of SVs | IGV verification |
| --- | --- |
| *lig4-4*: L1-4  Chr1: 6992317  69 bp deletion | 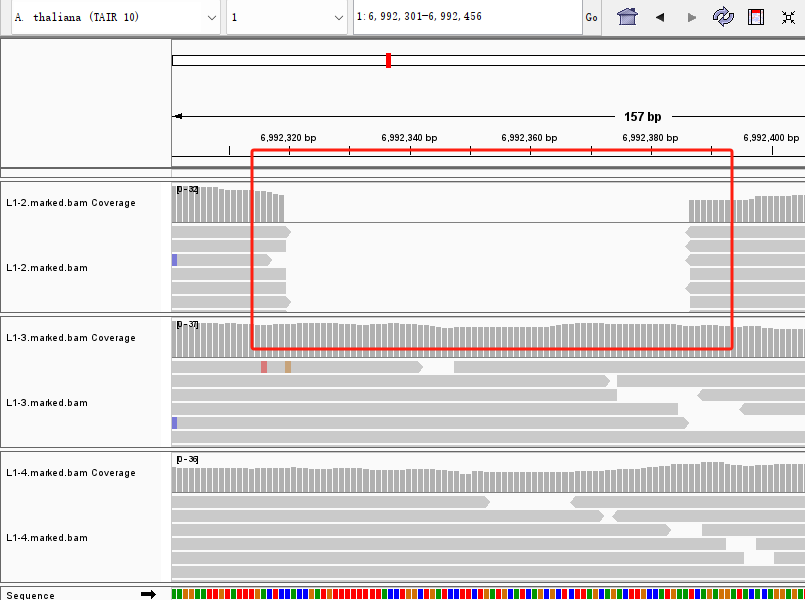 |
| *lig4-4*: L1-4  Chr1: 10993642  342 bp deletion | 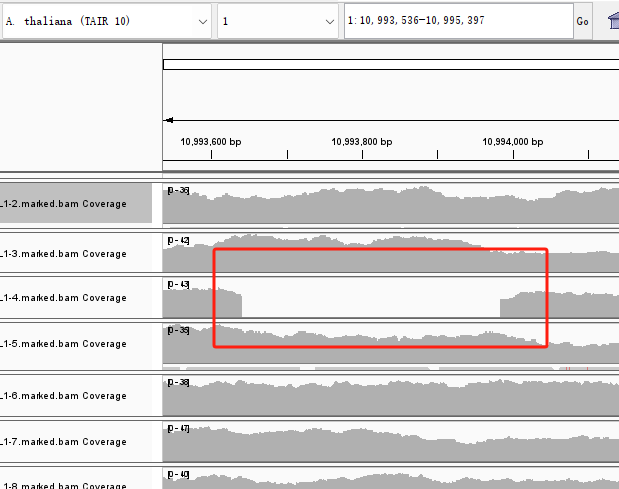 |
| *lig4-4*: L1-11  Chr1: 16939998  66 bp deletion | 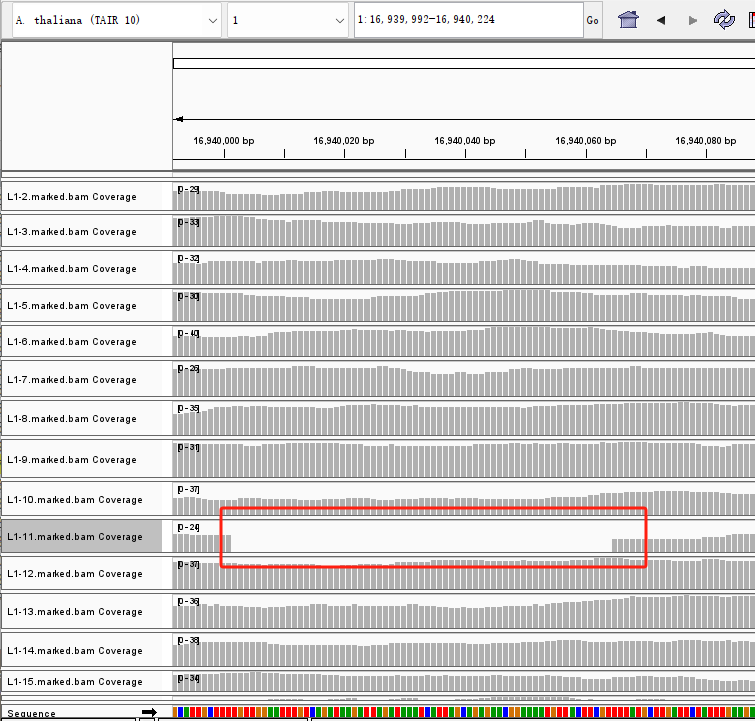 |
| *lig4-4*: L1-12  Chr1: 25618829  62 bp deletion | 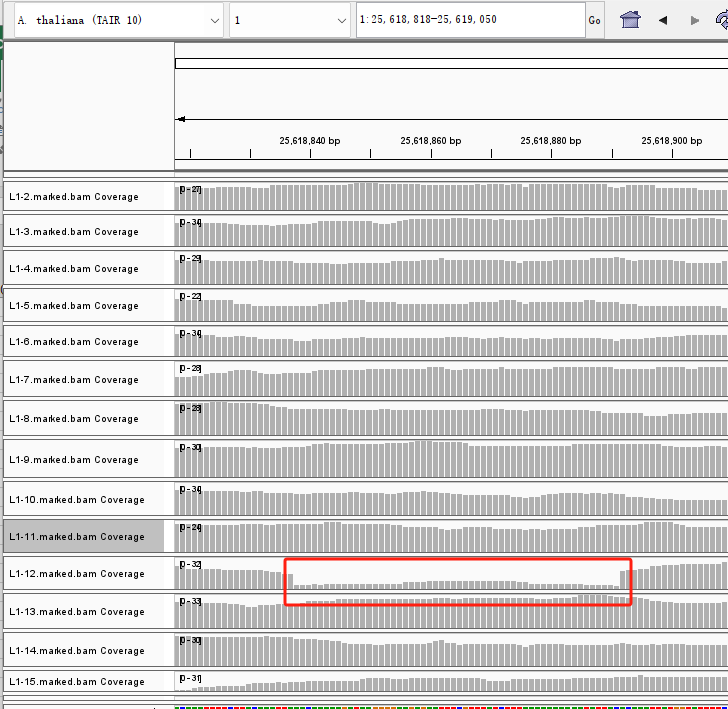 |
| *lig4-4*: L1-14  Chr1: 27307872  370 bp deletion | 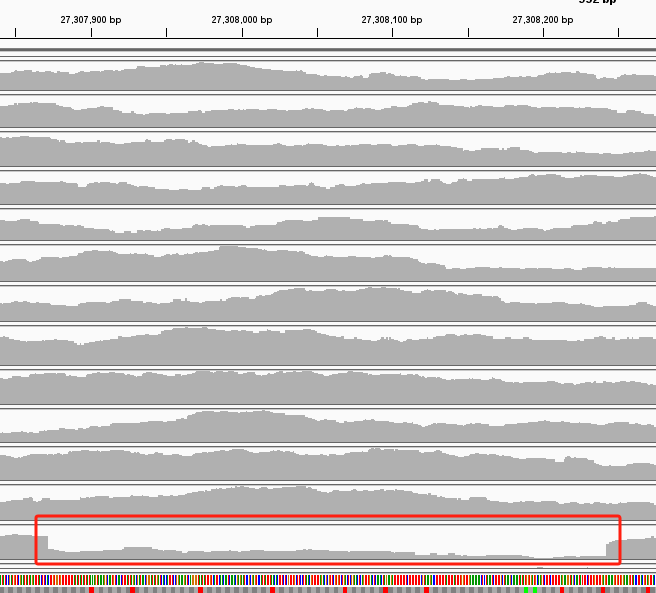 |
| *lig4-4*: L1-4  Chr1: 28491172  57 bp deletion | 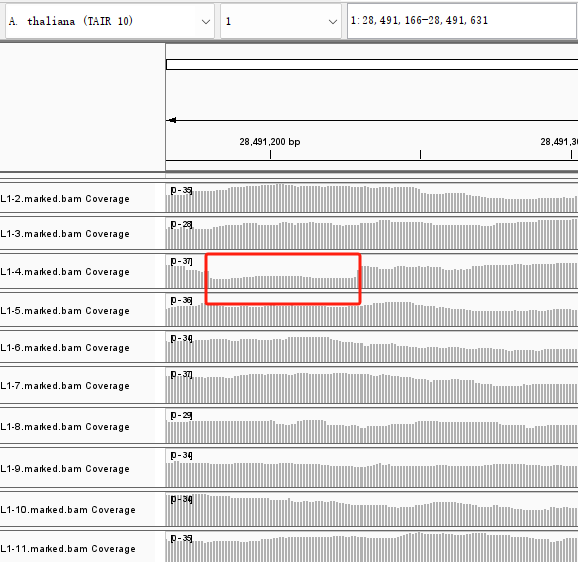 |
| *lig4-4*: L1-2  Chr1: 30164546  485 bp deletion | 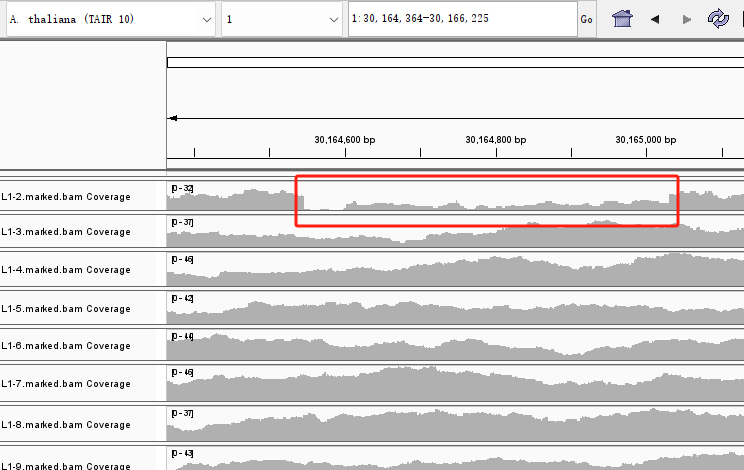 |
| *lig4-4*: L1-12  Chr2: 10395128  97 bp duplication | 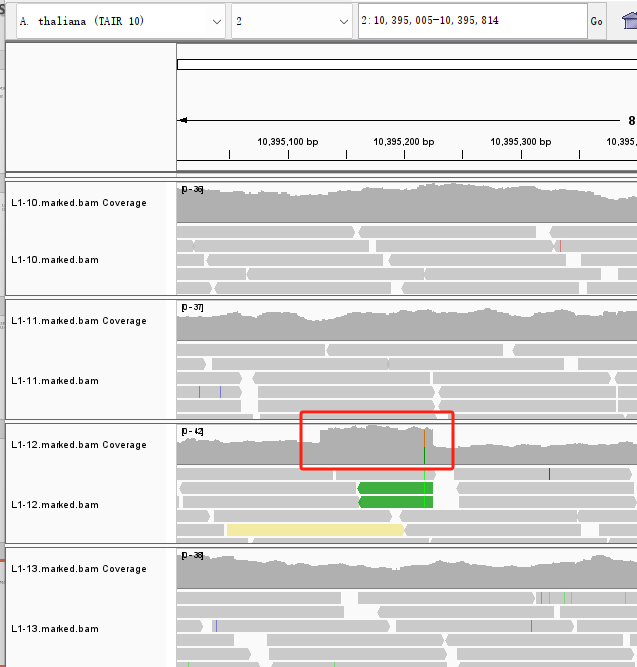 |
| *lig4-4*: L1-15  Chr3: 19928674  50 bp deletion | 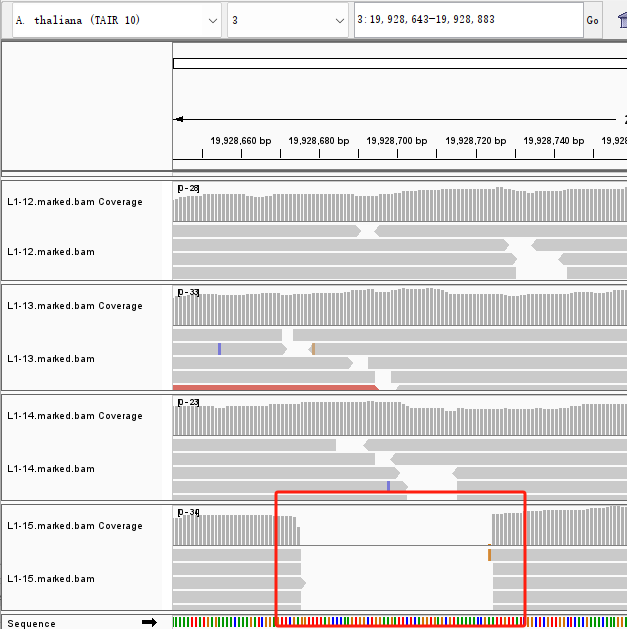 |
| *lig4-4*: L1-11  Chr3: 21916848  66 bp deletion | 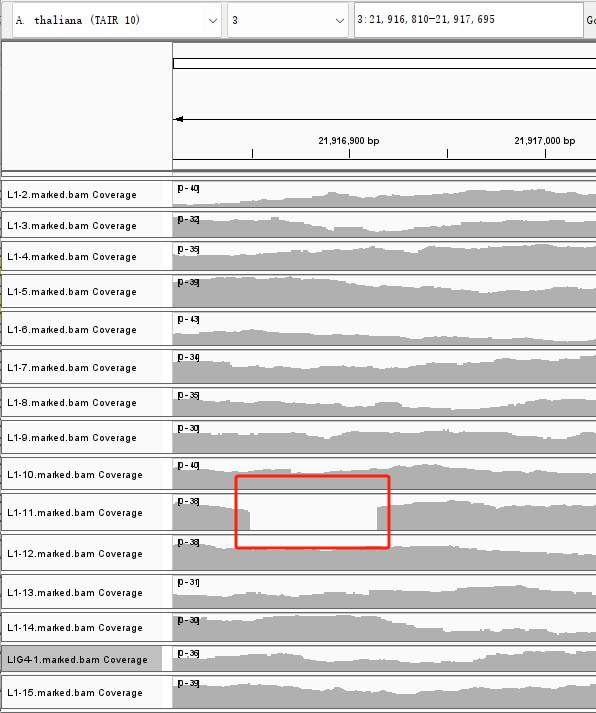 |
| *lig4-4*: L1-8  Chr4: 195851  86 bp deletion | 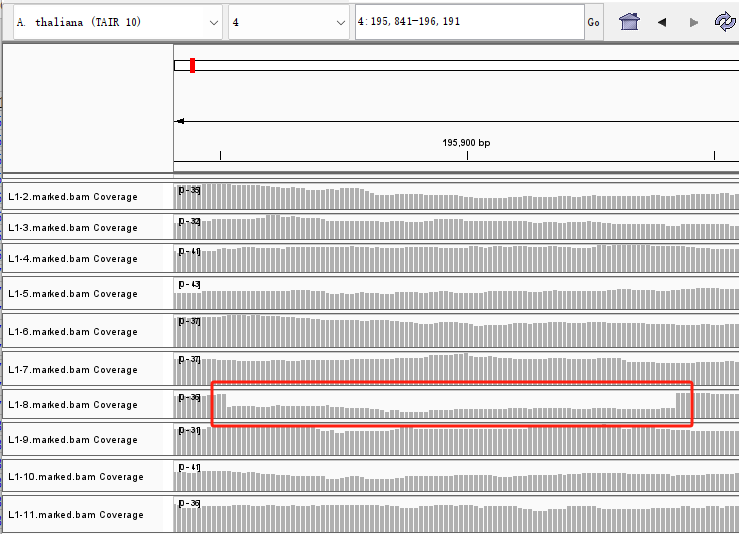 |
| *lig4-4*: L1-12  Chr4: 16040504  63 bp deletion | 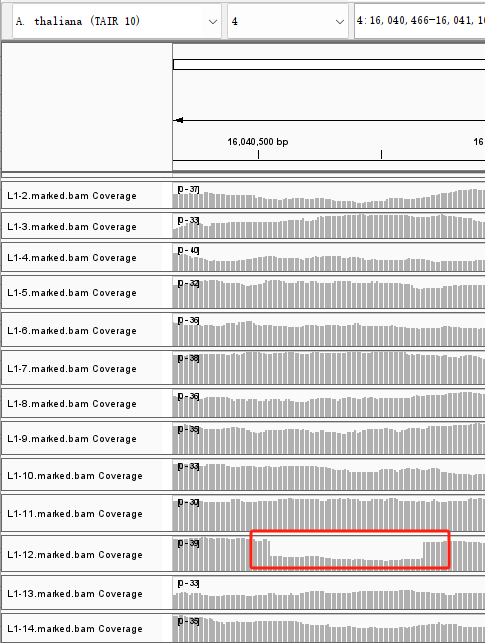 |
| *lig4-4*: L1-7  Chr4: 18179116  657 bp deletion | 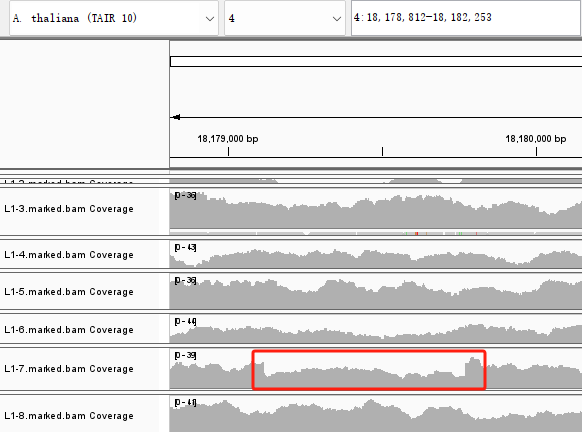 |
| *lig4-4*: L1-6  Chr4: 18289363  149 bp deletion | 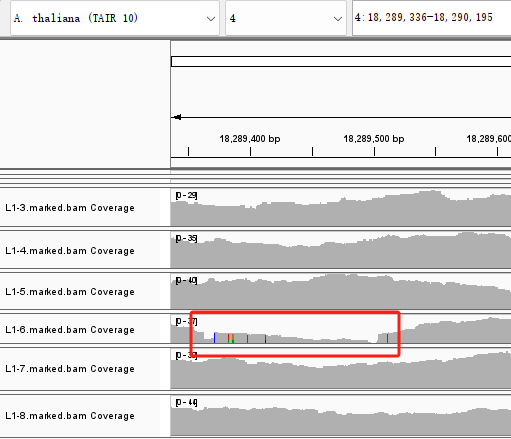 |
| *lig4-4*: L1-9  Chr5: 1745827  55 bp deletion | 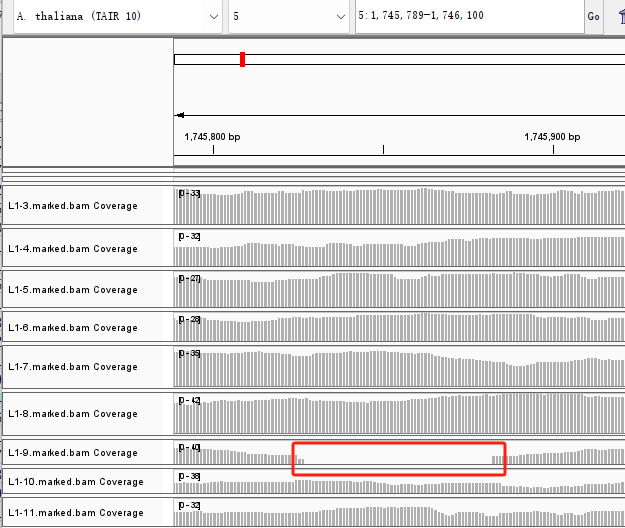 |
| *lig4-4*: L1-13  Chr5: 5531895  65 bp deletion | 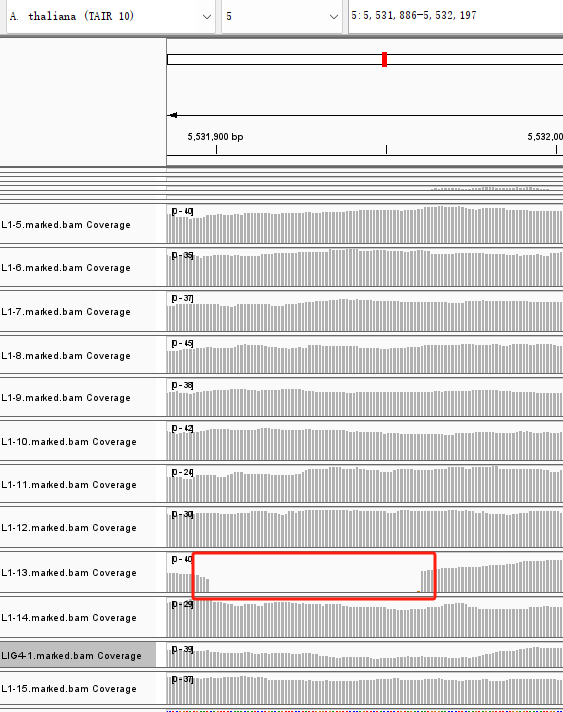 |
| *lig4-4*: L1-7  Chr5: 17879971  54 bp deletion | 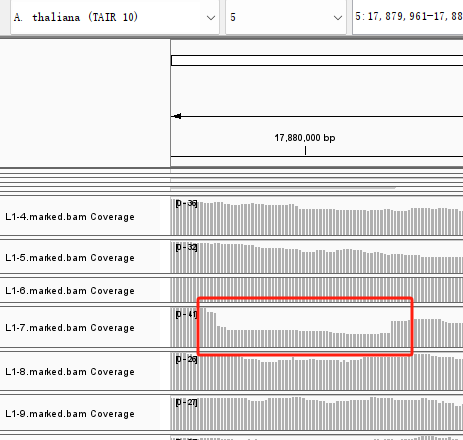 |
| *lig4-4*: L1-7  Chr5: 20646146  55 bp deletion | 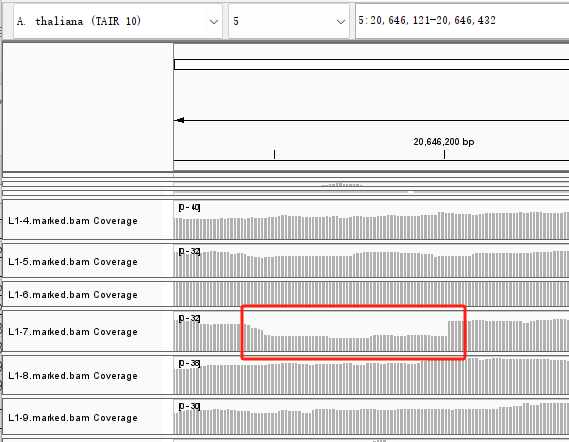 |
| WT: 3-4  Chr1: 21677471  61 bp deletion | 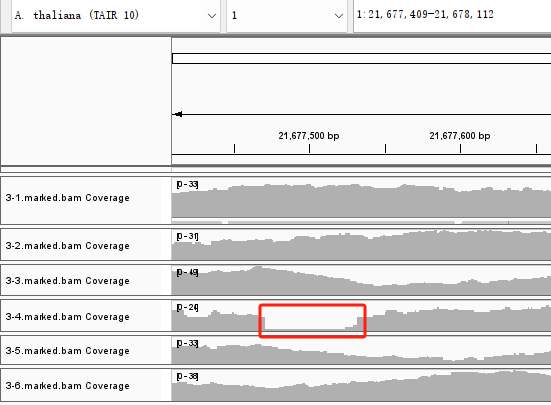 |
| WT: 3-4  Chr3: 307505  18588 bp deletion | 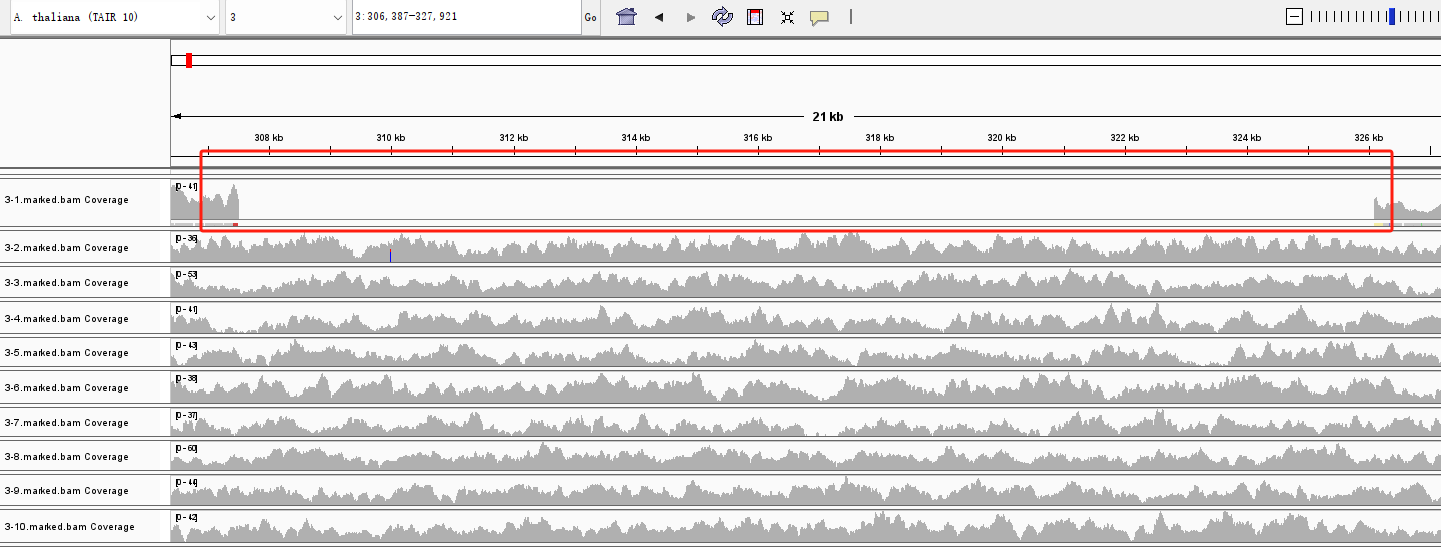 |

**Figure S7.** IGV visualization of SVs.
